# Microbiome dynamics and functional profiles in deep-sea wood-fall micro-ecosystem: insights into drive pattern of community assembly, biogeochemical processes, and lignocellulose degradation

**DOI:** 10.1128/aem.02165-24

**Published:** 2024-12-06

**Authors:** Zeming Bao, Biao Chen, Kefu Yu, Yuxin Wei, Xinyue Liang, Huanting Yao, Xianrun Liao, Wei Xie, Kedong Yin

**Affiliations:** 1 Guangxi Laboratory on the Study of Coral Reefs in the South China Sea, Coral Reef Research Center of China, School of Marine Sciences, Guangxi University 624446 , Nanning, China; 2 Southern Marine Science and Engineering Guangdong Laboratory (Zhuhai) 590852 , Zhuhai, China; 3 School of Marine Sciences, Sun Yat-sen University 626303 , Zhuhai, China; University of Delaware, Lewes, Delaware, USA

**Keywords:** deep-sea wood fall, microbial community assembly, biogeochemical process, cellulose metabolism, bacteria, fungi

## Abstract

Wood-fall micro-ecosystems contribute to biogeochemical processes in the oligotrophic deep ocean. However, the community assembly processes and biogeochemical functions of microbiomes in wood fall remain unclear. This study investigated the diversity, community structure, assembly processes, and functional profiles of bacteria and fungi in a deep-sea wood fall from the South China Sea using physicochemical indices, amplicon sequencing, and metagenomics. The results showed that distinct wood-fall contact surfaces exhibit habitat heterogeneity. The bacterial community of all contact surfaces and the fungal community of seawater contact surface (SWCS) were affected by homogeneous selection. In SWCS and transition region (TR), bacterial communities were influenced by dispersal limitation, whereas fungal communities were affected by homogenizing dispersal. The Venn diagram visualization revealed that the shared fungal community between SWCS and TR was dominated by Aspergillaceae. Additionally, the bacterial community demonstrated a higher genetic potential for sulfur, nitrogen, and methane metabolism than fungi. The sediment contact surface enriched modules were associated with dissimilatory sulfate reduction and methanogenesis, whereas the modules related to nitrate reduction exhibited enrichment characteristics in TR. Moreover, fungi showed a stronger potential for lignocellulase production compared to bacteria, with Microascaceae and Nectriaceae identified as potential contributors to lignocellulose degradation. These results indicate that environmental filtering and organism exchange levels regulated the microbial community assembly of wood fall. The biogeochemical cycling of sulfur, nitrogen, and methane was mainly driven by the bacterial community. Nevertheless, the terrestrial fungi Microascaceae and Nectriaceae might degrade lignocellulose via the combined action of multiple lignocellulases.

**IMPORTANCE:**

The presence and activity of microbial communities may play a crucial role in the biogeochemical cycle of deep-sea wood-fall micro-ecosystems. Previous studies on wood falls have focused on the microbiome diversity, community composition, and environmental impact, while few have investigated wood-fall micro-ecosystems by distinguishing among distinct contact surfaces. Our study investigated the microbiome dynamics and functional profiles of bacteria and fungi among distinct wood-fall contact surfaces. We found that the microbiome community assembly was regulated by environmental filtering and organism exchange levels. Bacteria drive the biogeochemical cycling of sulfur, nitrogen, and methane in wood fall through diverse metabolic pathways, whereas fungi are crucial for lignocellulose degradation. Ultimately, this study provides new insights into the driving pattern of community assembly, biogeochemical processes, and lignocellulose degradation in the microbiomes of deep-sea wood-fall micro-ecosystems, enhancing our comprehension of the ecological impacts of organic falls on deep-sea oligotrophic environments.

## INTRODUCTION

Extreme climatic events (e.g., floods and typhoons) (1, 2) and anthropogenic activities (e.g., shipwrecks and deforestation) (3, 4) can transport terrestrial wood debris into the ocean. Once waterlogged, the wood debris sinks to the seabed, promptly establishing a wood-fall micro-ecosystem that serves as a habitat for chemosynthetic organisms in the deep sea (5). In contrast to hydrothermal vents, cold seeps, and seamounts, wood falls are transient, island-like habitats with irregular spatial and temporal distribution (3, 5). The deep seafloor is characterized by extreme oligotrophy due to limited energy and nutrient influx (6, 7). In such conditions, organic falls, such as wood and whale falls, serve as essential energy sources for benthic ecosystems (8, 9). The settling of organic matter on nutrient-poor seafloors can rapidly create biodiversity hotspots (10), facilitating the formation of highly productive ecosystems by attracting benthic animals and microorganisms (11). In addition, the decomposition of wood and woody debris on the seafloor is a major ecological process in wood falls, primarily driven by benthic organisms and microorganisms (12). Degradation rates on different contact surfaces of wood are not synchronized, as they depend on immersion duration and the surrounding biomass (13–15). Moreover, anaerobic degradation of wood supports chemoautotrophic species and creates habitat heterogeneity on the deep seafloor, which can persist for extended periods (15, 16), and contributes to maintaining species diversity in wood-fall micro-ecosystems (17). Therefore, wood falls are vital sources of material and energy in deep-sea oligotrophic environments, offering an ideal system for investigating microbial community assembly processes and ecological functions.

Exploring community assembly processes is crucial for enhancing our understanding of the composition, structure, and function of microbial communities on wood falls (18). Deterministic and stochastic processes are the two key ecological mechanisms that govern microbial community assembly (19, 20). Deterministic processes are mainly the result of environmental and biological filtering (e.g., interspecific competition and intraspecific functional trait variation), including homogeneous and heterogeneous selection. In contrast, stochastic processes, including dispersal limitation, homogenizing dispersal, and drift, primarily reflect random fluctuations in species relative abundance (21). Environmental filtering has been proposed as a key factor influencing the structure of microbial communities in sunken wood (8). However, its significance varies with both time and microhabitats (22), suggesting that the effects of environmental filtering on microbial communities may differ across distinct contact surfaces of wood falls. Despite this, our knowledge about the driving patterns of microbial community assembly at distinct contact surfaces of wood-fall micro-ecosystems remains limited. Therefore, distinguishing among different contact surfaces to explore the underlying mechanisms of microbiome community assembly could provide a foundation for further assessing the enrichment effects of wood-fall micro-ecosystems on surrounding microorganisms.

The efficiency of wood degradation has a closed association with the presence and activity of microbial communities, which may further affect biogeochemical cycling within wood-fall micro-ecosystems (23), including the cycling of carbon, sulfur, and nitrogen. Wood-fall microbial communities typically consist of two main components: a wood-degrading heterotrophic community and a chemosynthetic community. Carbon in wood exists mainly in the form of lignocellulose, which is composed of cellulose, hemicellulose, and lignin (24). Of these, hemicellulose and cellulose are more easily degraded, whereas lignin is more resistant to degradation and difficult to break down into simpler compounds (25). Wood-degrading microorganisms in wood-fall decompose lignocellulose in wood (26, 27), while chemoenergetic microorganisms utilize the by-products of wood degradation, such as H₂S or CH₄, as energy sources (5, 12, 16). Moreover, due to the extremely low nitrogen content of wood (28), certain microorganisms in wood fall may exhibit strong nitrogen-fixing capabilities (29), allowing them to survive under nitrogen-limited conditions. However, the contribution of specific microorganisms on biogeochemical processes and lignocellulose degradation in wood-fall micro-ecosystems remains unclear. As global warming progresses, the intensity and frequency of extreme climatic events are projected to increase (30), potentially leading to more frequent deep-sea wood-fall events. Therefore, exploring how the wood-fall microbiome drives biogeochemical cycling and lignocellulose degradation is crucial for revealing the ecological functions of wood-fall micro-ecosystems.

**Fig 1:**
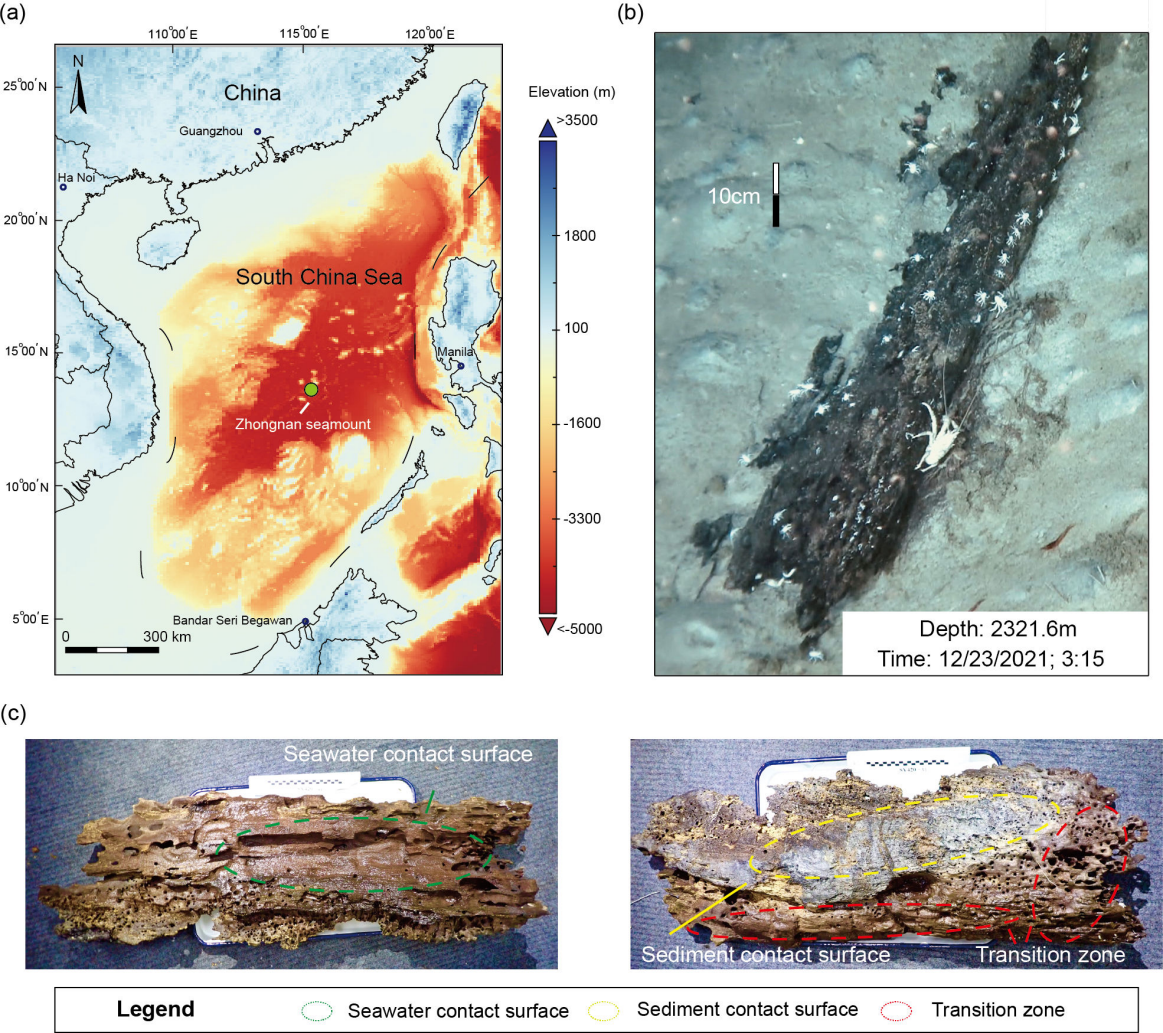
Sampling locations and distinct contact surfaces of deep sea wood fall. (**a**) Sampling location of deep-sea wood fall in the northern slope of Zhongnan seamount in the SCS. (**b**) *In situ* imaging of deep-sea wood fall was generated by the human-occupied vehicle “*Shenhaiyongshi.*” (**c**) The distinct contact surfaces of the deep-sea wood fall. Sediment contact surface (SDCS), seawater contact surface (SWCS), and transition region (TR). The distinct contact surfaces of the wood-fall sample exhibited varying signs of decay. The SWCS appeared smoother, while the TR showed macroscopic shipworm burrows. In contrast, the SDCS displayed blackened areas, indicating significant sulfate reduction processes in that region. Therefore, it is presumed that the wood-fall sample was likely in the sulfophilic stage at the time of discovery.

This study was based on deep-sea wood fall collected by the manned submersible “*Shenhaiyongshi*” at the Zhongnan seamount in the South China Sea (SCS) in December 2021 (Fig. 1). Using amplicon and metagenomic sequencing, we analyzed the diversity, community composition, community structure, and biogeochemical functions of the wood-fall microbiome. Physicochemical indices were simultaneously combined to analyze the driving pattern of community assembly, biogeochemical processes, and lignocellulose degradation. Our results provide new insights into the microbiome dynamics and functional profiles of wood falls, contributing to a deeper understanding of the ecological impacts of wood-fall micro-ecosystems in deep-sea oligotrophic environments.

## RESULTS

### Physicochemical indices of wood fall

**Fig 2:**
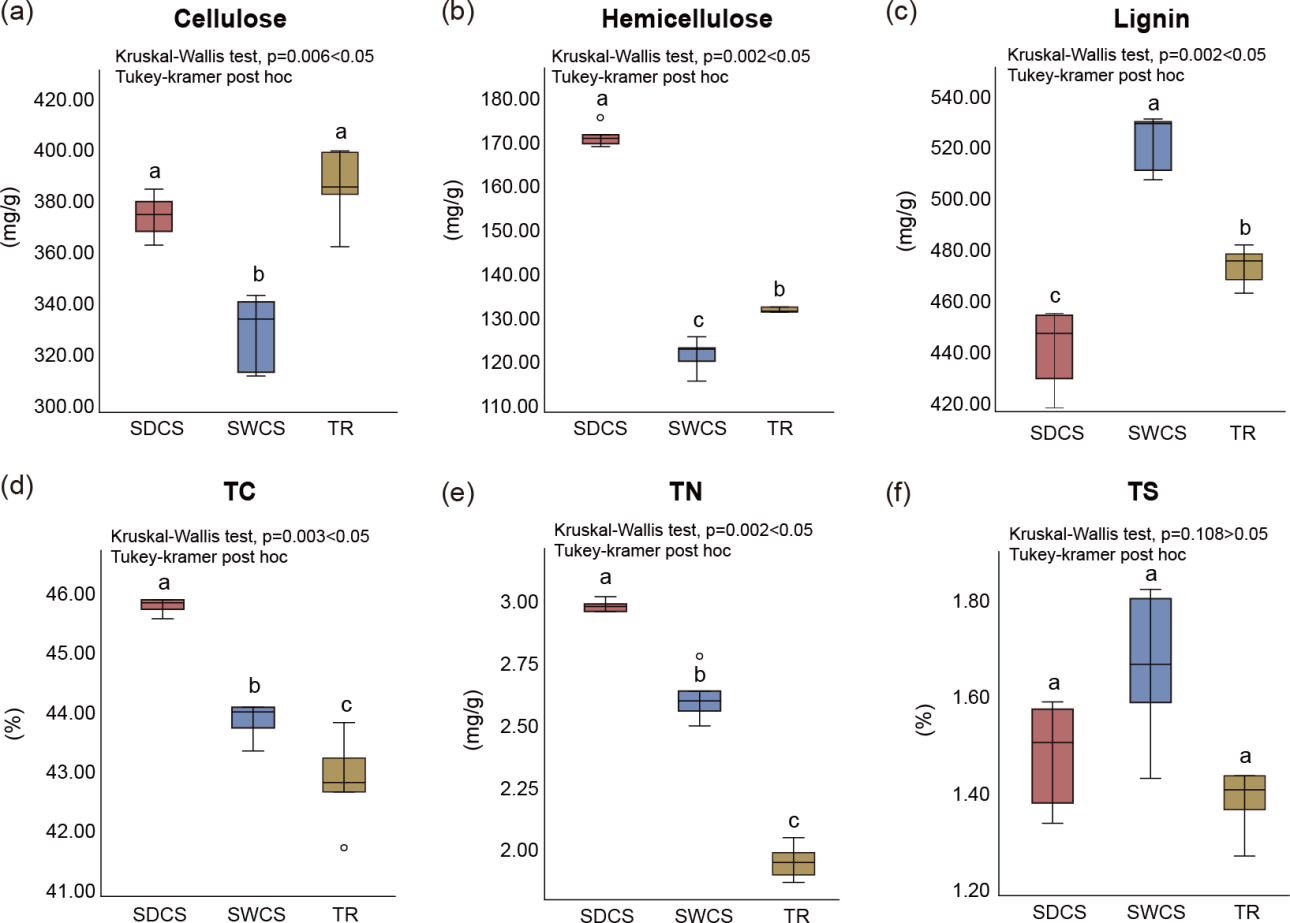
The physicochemical indices among distinct contact surfaces of wood fall. (**a**) Cellulose, (**b**) hemicellulose, (**c**) lignin, (**d**) total carbon (TC), (**e**) total nitrogen (TN), and (**f**) total sulfur (TS). Kruskal-Wallis test.

The analysis of physicochemical indices revealed significant differences among distinct contact surfaces of wood fall (Kruskal-Wallis H test, *P* < 0.05; Fig. 2; Table S1), with the exception of total sulfur (TS). Specifically, the contents of cellulose and hemicellulose were significantly lower in the seawater contact surface (SWCS) compared to both the sediment contact surface (SDCS) and the transition region (TR). Conversely, the lignin content was significantly higher in SWCS than in either SDCS or TR. Additionally, the total carbon (TC) and total nitrogen (TN) contents were significantly higher in SDCS than in SWCS and TR.

### Alpha diversity, community composition, and community structure of bacteria and fungi

**Fig 3:**
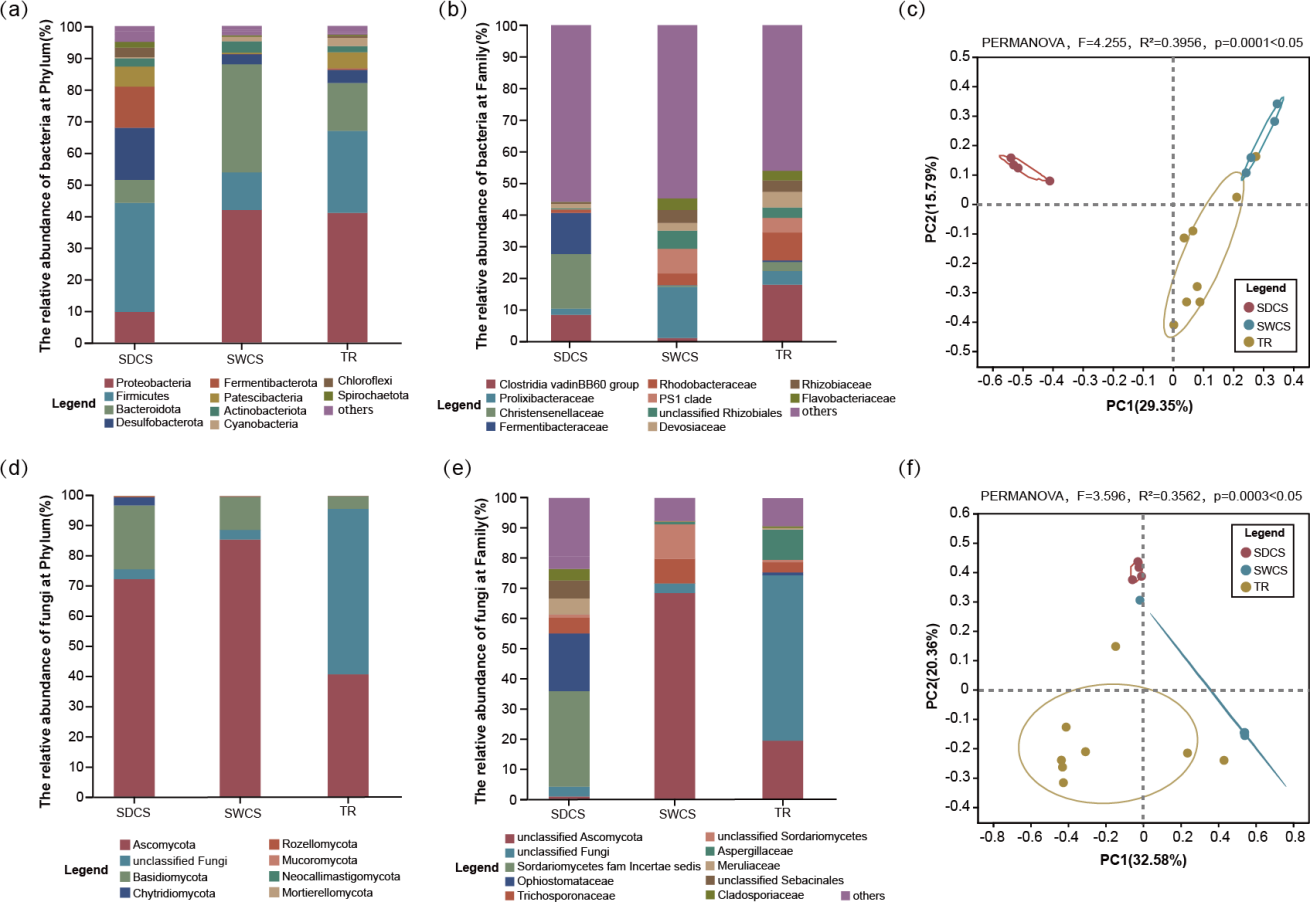
The bacterial and fungal community composition and structure among distinct contact surfaces of wood fall. (**a and b**) The relative abundance of bacteria at the phylum and family level. (**c**) The PCoA of Bray-Curtis distances of bacteria ASV compositions associated with three contact surfaces. (**d and e**) The relative abundance of fungi at the phylum and family level. (**f**) The PCoA of Bray-Curtis distances of fungi ASV compositions associated with three contact surfaces. Others represent the phyla and family with a relative abundance of less than 1%.

The results of the amplicon sequence variant (ASV) analyses showed that the 16S and ITS2 reads of wood fall could be clustered into 2,212 and 365 valid ASVs, respectively, after flattening the samples with a minimum number of sample sequences (bacteria: *n* = 18,528; fungi: *n* = 12,078), which were used in the subsequent assessment of differences in alpha diversity and community structure. Analyses of alpha diversity revealed no significant differences in the Shannon diversity index of bacteria (Kruskal-Wallis test, *P* = 0.597 > 0.05; Fig. S1a) or fungi (Kruskal-Wallis test, *P* = 0.189 > 0.05; Fig. S1b) among distinct contact surfaces (Table S2). At the phylum level, the bacterial community of wood fall was mainly dominated by the Proteobacteria (30.8% ± 18.3%), Firmicutes (24.1% ± 11.4%), Bacteroidota (18.8% ± 13.8%), and Desulfobacterota (7.9% ± 7.4%; Fig. 3a). At the family level, Clostridia vadin BB60 group (9.2% ± 8.4%), Prolixibacteraceae (7.5% ± 7.6%), and Christensenellaceae (6.9% ± 9.1%) dominated in the bacterial community (Fig. 3b). Notably, the types of bacteria that dominated the distinct contact surfaces were different. The bacterial community in SDCS had a higher abundance of Christensenellaceae (SDCS: 17.3%; SWCS: 0.5%; TR: 2.9%), and Prolixibacteraceae dominated the bacterial community of SWCS (SDCS: 2.0%; SWCS: 16.1%; TR: 4.4%), whereas that from TR was dominated by Clostridia vadin BB60 group (SDCS: 8.4%; SWCS: 1.2%; TR: 18%). In terms of fungi, the predominant fungal phyla were Ascomycota (66.2% ± 23.0%), unclassified Fungi (20.5% ± 29.9%), Basidiomycota (12.2% ± 8.5%), and Chytridiomycota (1.4% ± 1.9%; Fig. 3d). Ascomycota were ubiquitous and dominated fungal communities among distinct contact surfaces, with relative abundances ranging from 40.7% to 85.5%. However, the unclassified Fungi had a high relative abundance in TR (55.0%), whereas it was rare in SDCS (3.30%) and SWCS (3.20%). At the family level, the dominant fungi among distinct contact surfaces were unclassified Ascomycota (29.6% ± 34.9%), unclassified Fungi (20.5% ± 29.9%), Sordariomycetes fam *Incertae sedis* (10.6% ± 18.3%), and Ophiostomataceae (6.7% ± 10.8%; Fig. 3e). Ophiostomataceae was identified in SDCS (19.1%) and TR (0.9%) but not in SWCS and Sordariomycetes fam. *Incertae sedis* was only distributed in SDCS (31.8%). Furthermore, the results of principal co-ordinates analysis (PCoA) revealed significant differences in the community structure of bacteria (permutation multifactorial analysis of variance [PERMANOVA], *F* = 4.255, *R*^2^ = 0.3956, *P* = 0.0001 < 0.05; Fig. 3c) and fungi (PERMANOVA, *F* = 3.596, *R*^2^ = 0.3562, *P* = 0.0003 < 0.05; Fig. 3f) among distinct contact surfaces.

### Community assembly processes of bacteria and fungi

**Fig 4:**
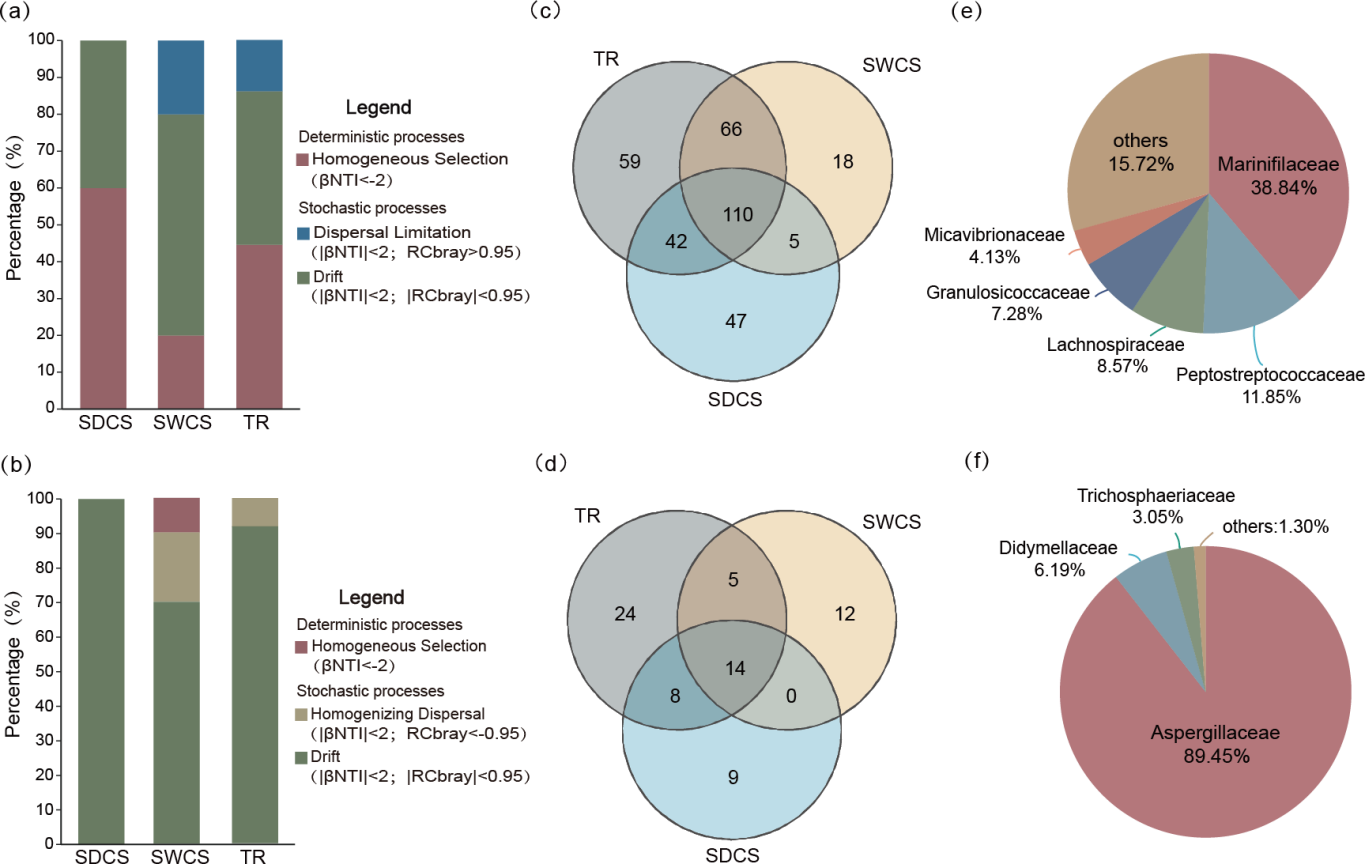
The community assembly process among distinct contact surfaces of wood fall and the shared microbiome composition of the bacteria and fungi among SWCS and TR at the family level. Contributions of deterministic processes and stochastic processes on (**a**) bacterial and (**b**) fungal community assembly processes. Venn diagrams at the family level showing the number of unique and shared (**c**) bacterial and (**d**) fungal families among the three contact surfaces of wood fall. The shared (**e**) bacterial families and (**f**) fungal families in SWCS and TR.

Bacterial community assembly in wood fall was influenced by both deterministic (41.5% ± 20.2%) and stochastic (58.5% ± 20.2%) processes, whereas the fungal community assembly was mainly controlled by stochastic processes (96.7% ± 5.8%; Fig. 4a and b). Specifically, the bacterial community was primarily affected by drift (SDCS: 40%; SWCS: 60%; TR: 41.67%), followed by homogeneous selection (SDCS: 60%; SWCS: 20%; TR: 44.4%), with dispersal limitation occurring only in SWCS (20%) and TR (13.89%). Drift was the most important assembly process for the fungal community (SDCS: 100%; SWCS: 70%; TR: 91.7%). Homogenized dispersal affected the fungal community only in SWCS (20%) and TR (8.33%), and only the fungal community structure in SWCS was affected by homogeneous selection. The shared and unique numbers of bacteria and fungi at the family level were identified using Venn diagrams. Venn diagram visualization revealed that 66 bacterial families and five fungal families were shared between SWCS and TR (Fig. 4c and d). Further analysis revealed that the shared community of bacteria was predominantly composed of Marinifilaceae (38.84%), Peptostreptococcaceae (11.85%), Lachnospiraceae (8.57%), and Granulosicoccaceae (7.28%; Fig. 4e), while the shared fungal community was dominated by Aspergillaceae (89.45%; Fig. 4f).

### Sulfur, nitrogen, and methane metabolism functions and the relative contribution of bacteria and fungi

**Fig 5:**
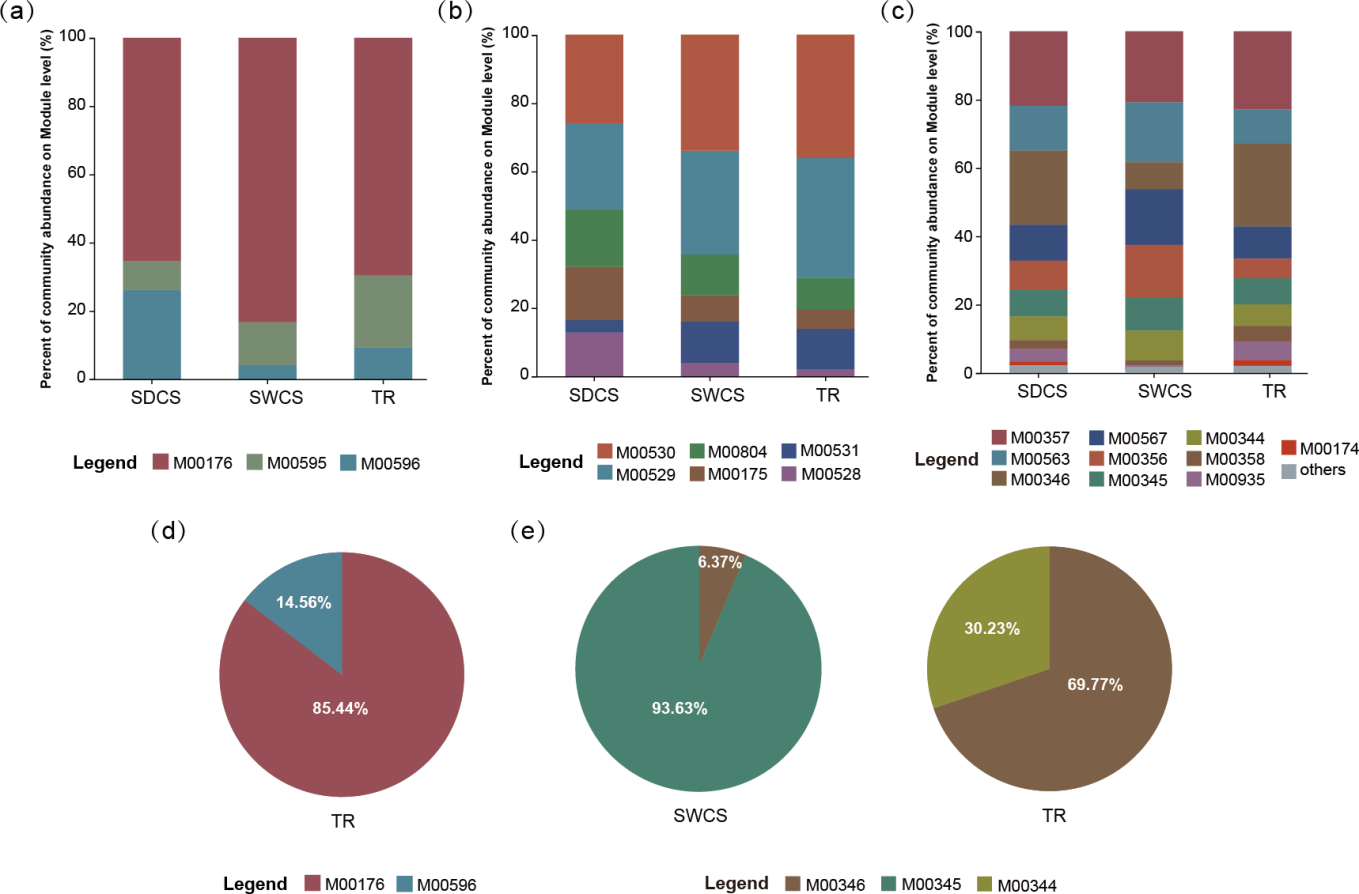
Functional characteristics of pathways for sulfur, nitrogen, and methane metabolism among distinct contact surfaces of wood fall. (**a**) Sulfur, (**b**) nitrogen, and (**c**) methane metabolism module in bacterial community; (**d**) sulfur and (**e**) methane metabolism module in fungal community. Others represent the modules with a relative abundance of less than 1%.

The Kyoto Encyclopedia of Genes and Genomes (KEGG) annotation identified 3 sulfur metabolism modules, 6 nitrogen metabolism modules, and 13 methane metabolism modules in bacterial genomes (Table S3). In contrast, fungal genomes exhibit two sulfur metabolism modules and three methane metabolism modules (Table S4). Selection modules with relative abundance of more than 1% in at least one wood-fall contact surface for visualization (Fig. 5; Table S5). For sulfur metabolism, M00176 (assimilatory sulfate reduction) exhibited the highest abundance in bacterial (72.8% ± 9.3%) and fungal (85.4%) communities (Fig. 5a and d). In nitrogen metabolism, M00530 (dissimilatory nitrate reduction; 32% ± 5.3%) and M00529 (denitrification; 30.2% ± 5.0%) were the primary modules involved in nitrogen metabolism in bacteria. As for methane metabolism, methanogenesis (M00357, M00563, M00567, and M00356) and formaldehyde assimilation (M00344, M00345, and M00346) were the primary pathways which bacteria potentially participate in methane metabolism, whereas only formaldehyde assimilation modules are detected in fungal communities.

**Fig 6:**
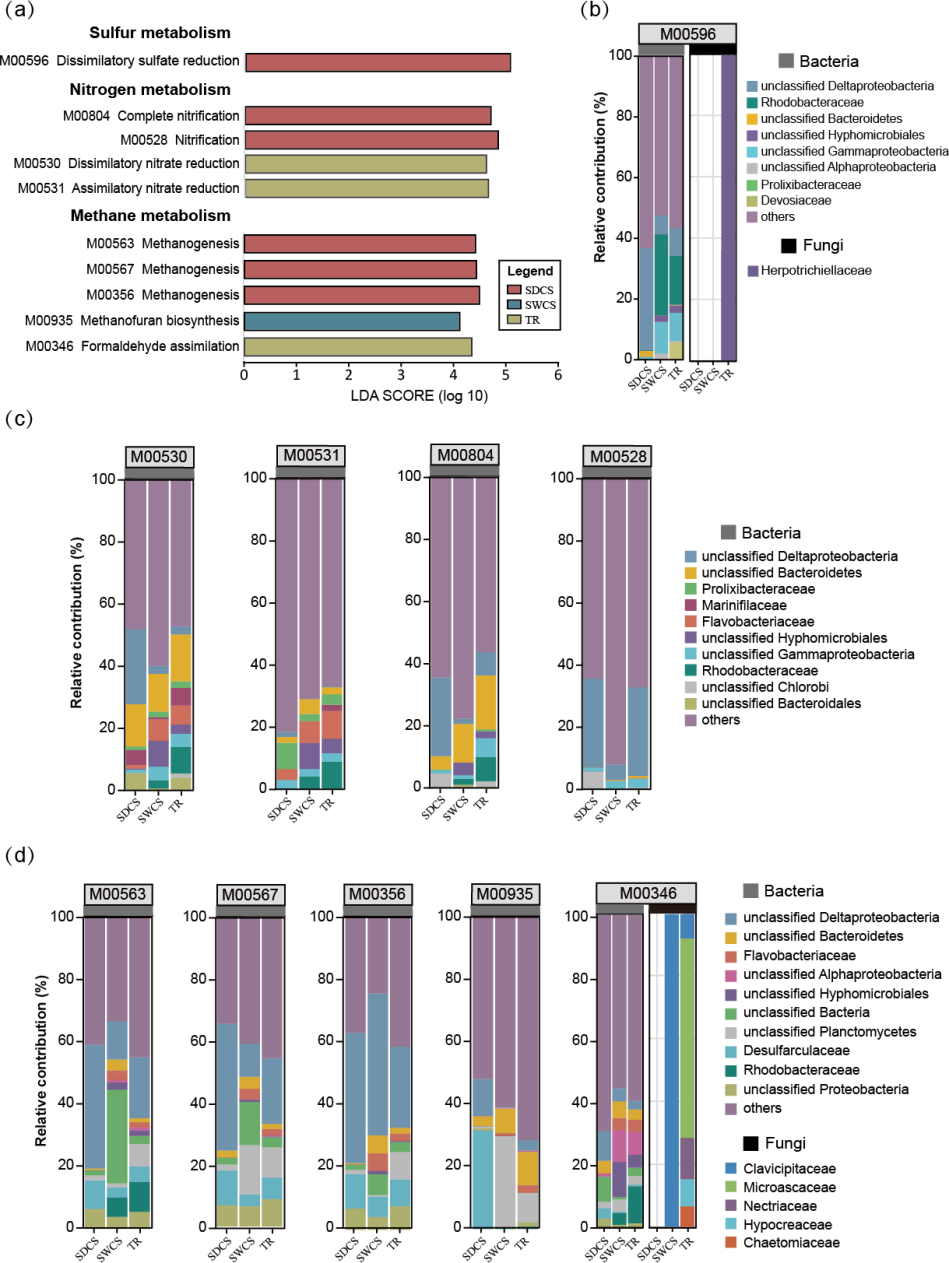
The enrichment characteristics and species contribution of the sulfur, nitrogen, and methane metabolism modules in microbiome of wood fall. (**a**) The enrichment modules of the sulfur, nitrogen, and methane metabolism in bacteria of wood fall (LDA > 4.0; *P* < 0.05). The relative contributions of bacteria and fungi (the family with the top 10 relative abundances) to (**b**) sulfur, (**c**) nitrogen, and (**d**) methane metabolism enrichment modules among distinct contact surfaces of wood fall.

Linear discriminant analysis (LDA) effect size (LEfSe) analysis demonstrated that a total of 10 modules associated with sulfur, nitrogen, and methane metabolism exhibited enrichment characteristics in the bacterial communities among distinct contact surfaces (LDA > 4; *P* < 0.05; Fig. 6a; Table S6), while no modules were enriched in fungal communities. Among them, SDCS enriched M00596 (dissimilatory sulfate reduction), M00528 (nitrification), M00804 (complete nitrification), and modules related to methanogenesis (M00563, M00567, and M00356). In addition, M00530, M00531, and M00346 were enriched in TR, whereas only M00935 (methanofuran biosynthesis) was enriched in SWCS. Moreover, we evaluated the completeness of the enriched sulfur, nitrogen, and methane metabolism modules within the bacterial community based on the KEGG orthology (KOs) information (Fig. S2; Table S3). The results indicate that the modules associated with dissimilatory sulfate reduction, nitrate reduction, methanofuran biosynthesis, and formaldehyde assimilation could be considered complete. In contrast, deletions of KOs were identified in pathways related to nitrification (M00528 and M00804) and methanogenesis (M00563, M00567, and M00356). Thus, the wood-fall bacterial communities may only participate in the intermediate steps of nitrification and methanogenesis.

To visualize the association between microbiomes and metabolic functions, the contributions of the top 10 families in relative abundance to sulfur, nitrogen, and methane metabolism modules enriched at distinct contact surfaces were chosen and analyzed. For sulfur metabolism (Fig. 6b), unclassified Deltaproteobacteria and Rhodobacteraceae were the main contributors to M00596. Specifically, unclassified Deltaproteobacteria made the highest contribution in SDCS (SDCS: 33.7%; SWCS: 6.2%; TR: 6.3%), whereas Rhodobacteraceae mainly contributed in SWCS and TR (SDCS: 0.1%; SWCS: 26.6%; TR: 13.5%). As for the fungi, Herpotrichiellaceae contributed 100% to M00596 in TR. In terms of nitrogen metabolism (Fig. 6c), the dominant contributors to M00530 were unclassified Bacteroidetes (contribution: 15.1%) and to M00531 were Flavobacteriaceae (contribution: 8.9%). The relative contributions of unclassified Deltaproteobacteria to M00528 and M00804 were 28.6% and 25.3%, respectively. For methane metabolism (Fig. 6d), unclassified Deltaproteobacteria was the main contributor to the intermediate processes of methanogenesis in wood fall, and its relative contribution to these methane metabolism modules ranged from 23.7% to 37.5%. Furthermore, M00935 was mainly contributed by Desulfarculaceae (SDCS: 31.42%; SWCS: 0.24%; TR: 0.82%) and unclassified Planctomycetes (SDCS: 0.65%; SWCS: 28.96%; TR: 9.33%). For M00346, Rhodobacteraceae made the most significant contribution in TR (SDCS: 0.02%; SWCS: 3.76%; TR 11.77%). Notably, the fungal community consisting of Clavicipitaceae, Microascaceae, Nectriaceae, Hypocreaceae, and Chaetomiaceae contributed to M00346.

### Lignocellulose degradation function and the relative contribution of bacteria and fungi

**Fig 7:**
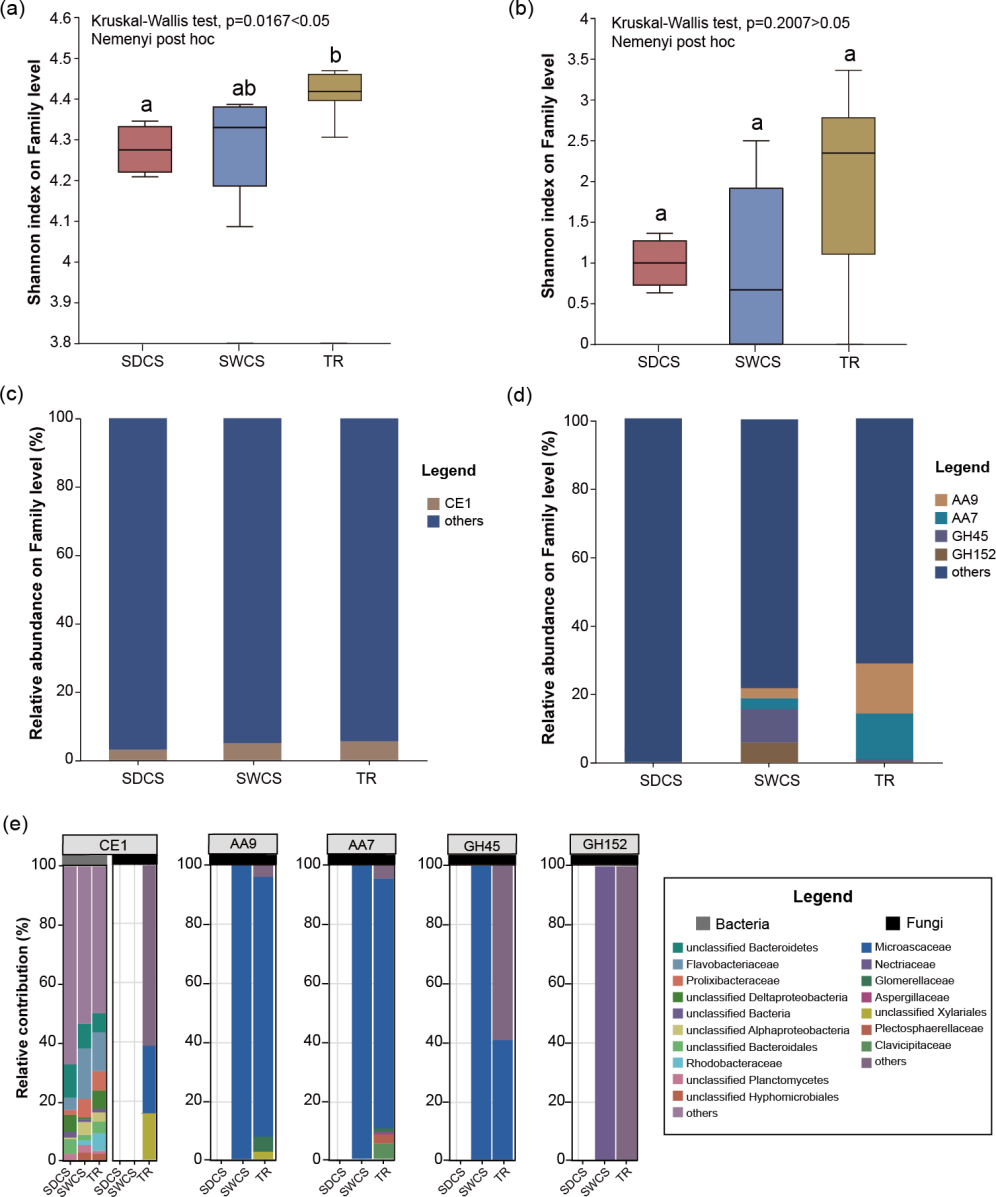
The lignocellulose degradation function and the relative contribution of bacteria and fungi in wood fall. The Shannon diversity index of (**a**) bacteria and (**b**) fungi CAZymes gene among distinct contact surfaces of wood fall. Relative abundance of the families containing lignocellulases of (**c**) bacteria and (**d**) fungi among distinct contact surfaces of wood fall. Others represent the families with a relative abundance of less than 5% in each contact surface of wood fall. (**e**) The relative contributions of bacteria and fungi (the family with the top 10 relative abundances) to the families containing lignocellulases among distinct contact surfaces of wood fall.

Analyses of α diversity showed that the Shannon diversity index of the bacterial CAZymes gene at family levels in TR was significantly higher than that in SDCS (Kruskal-Wallis test, *P* = 0.0167 < 0.05; Fig. 7a), while the Shannon diversity index of the fungal CAZymes gene at family levels did not differ significantly among distinct contact surfaces (Kruskal-Wallis test, *P* = 0.2 > 0.05; Fig. 7b). Families that contain lignocellulases and with a relative abundance >5% in at least one contact surfaces were selected to perform functional composition analysis. For bacterial community, only CE1 met the screening criteria, and its relative abundance among distinct contact surfaces ranged from 3.0% to 5.5% (Fig. 7c; Table S7). In terms of fungal community, a total of four families were identified that met the criteria and were only present in SWCS and TR (Fig. 7d; Table S7). Among these, GH45 (SWCS: 9.70%; TR: 1.09%) and GH152 (SWCS: 5.97%; TR: 0.30%) exhibited higher abundances in SWCS, whereas the TR was dominated by AA9 (SWCS: 2.88%; TR: 14.48%) and AA7 (SWCS: 3.18%; TR:13.22%). Thus, wood-fall fungi have a higher genetic potential for lignocellulose degradation than bacteria, and SWCS and TR may be the main regions where fungi secrete lignocellulases. Species and functional contribution analyses showed that Flavobacteriaceae and unclassified Bacteroidetes were the primary potential contributors to CE1, with relative contributions of 11.5% ± 6.6% and 8.8% ± 2.4%, respectively. Notably, Microascaceae (contribution: 22.9%) and unclassified Xylariales (contribution: 15.8%) also contributed to CE1 in TR. Additionally, Microascaceae and Nectriaceae were the primary contributing fungi for lignocellulose degradation. Of these, Microascaceae was the primary contributor to the AA9, AA7, and GH45 families, accounting for 100% of these families in SWCS. In TR, Microascaceae contributed 88.3%, 85.0%, and 40.7% to these families, respectively. The relative contribution of Nectriaceae to GH152 was 100%. Moreover, fungi with lignocellulose degradation potential in wood-fall micro-ecosystem also included Clavicipitaceae, Plectosphaerellaceae, Glomerellaceae, unclassified Xylariales, and Aspergillaceae.

## DISCUSSION

### Environmental filtering and organism exchange levels regulated the microbial community assembly in deep-sea wood-fall micro-ecosystem

Physiochemical analyses revealed significant differences in physicochemical indices across distinct contact surfaces, with the exception of TS. PCoA further showed significant differences in the bacterial and fungal community structures among distinct contact surfaces. Homogeneous selection influenced the bacterial communities among the three contact surfaces, but it only occurred in the fungal community of SWCS. These results demonstrate that distinct contact surfaces of wood fall exhibit habitat heterogeneity, which in turn shapes the assembly processes of microbial communities. This may result from varying sensitivities of bacteria and fungi to environmental filtering within wood-fall micro-ecosystem. Previous studies have indicated that non-homogeneous wood degradation can create multiple micro-niches, decrease competition among community members (8), and thereby facilitate species colonization and coexistence within communities (31, 32). Thus, habitat heterogeneity may enhance the impact of environmental filtering on regulating the community structure of microbiomes among differential contact surfaces of wood fall (33). In addition, Kalenitchenko et al. (22) demonstrated that only specific microorganisms are able to colonize and persist in wood substrates during the early stages of microbial community assembly, illustrating that environmental filtering plays a selective role in the microbial community. In this study, physicochemical indices on the same contact surface demonstrated greater homogeneity compared to those between the distinct contact surfaces, which may be the primary reason for the influence of homogeneous selection on microbial community assembly in wood-fall ecosystems. However, the observed differences in homogeneous selection between bacterial and fungal communities suggest that bacterial communities may be more sensitive to environmental changes among distinct contact surfaces in wood-fall micro-ecosystem.

In terms of stochastic processes, dispersal limitation and homogenizing dispersal were observed only in SWCS and TR, respectively, affecting the assembly processes of bacterial and fungal communities. The Venn diagram visualization revealed that the shared fungal community between SWCS and TR was predominantly composed of Aspergillaceae. Previous studies have shown that the level of environmental filtering decreases over time (22), which establishes a foundation for microbial migration. However, the theory proposed by Stegen et al. (34) suggests that dispersal limitation arises from limited organism exchange among communities, whereas homogenizing dispersal occurs when there are high levels of organism exchange. These results imply that dispersal limitation and homogenizing dispersal may be driven by differences in organism exchange levels within the shared bacterial and fungal communities of SWCS and TR. Previous research has indicated that the *Aspergillus* (Aspergillaceae) is widely distributed in marine environments and secretes a variety of secondary metabolites with antibacterial activities (35, 36). For example, Xiang et al. (37) reported that secondary metabolites from *Aspergillus flavus* SCSIO F025, isolated from deep-sea sediments in the SCS, exhibit antimicrobial activity against various bacteria. Similarly, *Aspergillus jensenii* LW128, isolated from deep-sea sediments in the northwestern Pacific Ocean, produces secondary metabolites with broad-spectrum antibacterial activity, inhibiting the growth of bacteria such as *Escherichia coli* and *Bacillus* species (38, 39). Thus, the dominant Aspergillaceae within the shared microbial communities might inhibit the growth and dispersal of bacteria by secreting secondary metabolites with bacteriostatic activities, leading to a restricted exchange of bacterial communities between SWCS and TR. Additionally, as terrestrial plant pathogens, Aspergillaceae exhibit extremotolerance and grow on a wide range of carbon sources (40). For instance, Damare et al. (41) found that spores of a deep-sea isolate of *Aspergillus terreus* were capable of germinating at 200 bar, confirming that terrestrial fungi can adapt to the extreme conditions of the deep sea and remain viable in high-pressure environments. Furthermore, some *Aspergillus* species were characterized by high conidia production, rapid growth, and distributions that support the “everything is everywhere” theory (42, 43). Therefore, Aspergillaceae could achieve a high level of organism exchange in SWCS and TR due to their strong adaptability and dispersal capability.

Our findings indicate that both environmental filtering and organism exchange levels shape the microbial community assembly in deep-sea wood-fall micro-ecosystems. Distinct contact surfaces of wood fall exhibit habitat heterogeneity, which causes a strong environmental filtering process. Stable environmental factors within the same contact surface resulted in microbial community assembly influenced by homogeneous selection. However, bacteria and fungi among distinct contact surfaces may display different sensitivities to habitat heterogeneity. The appearance of dispersal limitation and homogenizing dispersal in SWCS and TR might be associated with variations in organism exchange level between bacteria and fungi within the shared microbial communities.

### Bacteria drive the biogeochemical cycling in deep-sea wood-fall micro-ecosystem through diverse metabolic pathways

Functional composition analyses revealed that bacteria displayed a higher metabolic potential for sulfur, nitrogen, and methane metabolism, whereas fungi displayed limited pathways for these processes, restricted to specific contact surfaces in wood fall. Additionally, 10 modules related to sulfur, nitrogen, and methane metabolism were enriched in bacterial communities of SDCS and TR, with no enrichment observed in fungal communities. These results indicated that bacteria may drive biogeochemical cycling in wood-fall micro-ecosystems through diverse metabolic pathways. Primary production in deep-sea chemosynthetic ecosystems is driven by chemosynthetic communities that rely on reduced compounds, such as sulfide and methane (44). The sulfur cycle plays a crucial role in driving microbial life and biogeochemical cycling on the seafloor and is tightly interwoven with other important element cycles, such as carbon and nitrogen (45). Previous studies have suggested that bacteria alone can establish the chemical basis of wood-fall micro-ecosystems by facilitating the development of sulfidic niches (5, 12). In this study, the enrichment of M00596 in SDCS indicates that bacteria have the potential to produce a large amount of hydrogen sulfide in this area through the process of dissimilatory sulfate reduction. In contrast, in SWCS and TR, sulfide production is sustained by the degradation of lignocellulose in wood, which provides low molecular weight organic compounds to sulfate-reducing prokaryotes (46). Therefore, highly efficient sulfate reduction processes occur in all contact surfaces of wood fall, which may explain the lack of significant differences in the TS content among distinct contact surfaces. Notably, unclassified Deltaproteobacteria contributed the most to M00596 in SDCS, possibly because most sulfate-reducing bacteria are members of the Deltaproteobacteria (47). Additionally, the TC level in SDCS was significantly higher than that in both SWCS and TR, and the Shannon diversity of CAZyme genes in bacterial communities was significantly higher in TR compared to SDCS. As the degradation of wood can lead to carbon enrichment in the surrounding sediment of wood falls (15), the SDCS of wood-fall micro-ecosystems can be considered a high-carbon environment. It is believed that sulfate reducers and methanogens are spatially or temporally separated from each other (48). In marine environments, sulfate reduction is preferred over methanogenesis for the degradation of organic matter (48, 49). Nevertheless, Fagervold et al. (8) demonstrated the first evidence of the co-occurrence of sulfate-reducing bacteria and methanogens in wood-fall micro-ecosystems, suggesting that sulfate reduction and methanogenesis can occur simultaneously in high-carbon environments (50, 51). This was supported by the simultaneous enrichment of modules related to sulfate reduction (M00596) and methanogenesis (M00563, M00567, and M00356) in SDCS in our study. However, metabolic pathway maps of enriched modules indicate that bacteria have the potential to participate only in intermediate steps of methanogenesis, as they lack the Methyl-coenzyme M reductase (EC 2.8.4.1), which catalyzes the final step of methanogenesis and mediated only by methanogenic archaea (52, 53).

As for nitrogen cycling, the results showed that bacteria can drive nitrogen cycling through a variety of pathways, including nitrate reduction, nitrification, denitrification, and nitrogen fixation. Among them, M00530 and M00529 dominated the distinct contact surfaces, suggesting that both closed and open nitrogen cycling exist in wood-fall micro-ecosystems. The LEfse analysis revealed that M00530 and M00531 were differentially enriched in TR, whereas M00528 and M00804 exhibited enrichment characteristics in SDCS. Given that the TN content of SDCS was significantly higher than that of TR, the TR can be considered a nitrogen-limited area. Thus, these results imply that under nitrogen-limited conditions, bacteria tend to engage in closed nitrogen cycling in wood-fall micro-ecosystems. In natural ecosystems, denitrification and dissimilatory nitrate reduction compete with nitrate as an electron acceptor (54). Unlike denitrification, dissimilatory nitrate reduction preserves bioavailable nitrogen in the system and produces soluble ammonium instead of unreactive dinitrogen gas (55). Consequently, nitrate reduction can prevent nitrogen loss and is important for maintaining nitrogen levels and primary productivity in wood-fall micro-ecosystems. Thus, a closed nitrogen cycle facilitates the efficient conservation and recycling of nitrogen in wood-fall micro-ecosystems.

Accordingly, bacteria serve as the primary drivers of sulfur, nitrogen, and methane metabolism in wood-fall micro-ecosystems and are essential for facilitating biogeochemical cycling in wood-fall micro-ecosystems through their diverse metabolic pathways. While both sulfate reduction and methanogenesis can occur in high-carbon environments (SDCS), bacteria possess only the genetic potential to participate in intermediate steps of methanogenesis. Under nitrogen-limited conditions (TR), the bacterial community tends to maintain nitrogen levels through a closed nitrogen cycle.

### Terrestrial fungi may efficiently degrade cellulose and hemicellulose in the wood-fall micro-ecosystem by secreting diverse lignocellulases

The CAZy annotation results revealed that the fungal community could secrete a wider range of lignocellulases than the bacterial community. Four families within the fungal community were identified as possessing lignocellulases, whereas bacteria secreted lignocellulases only through CE1 family. This suggests that lignocellulose degradation in wood-fall micro-ecosystems may potentially be driven by fungi. It is widely known that fungi have the ability to utilize a broad spectrum of organic substrates for growth and can degrade a variety of organic substrates in deep sub-seafloor sediments (56, 57). Since most lignocellulases are derived from fungi (58, 59), they are consequently major decomposers of woody substrata in marine ecosystems (60) and possibly play a crucial role in the decomposition of wood substrates in deep-sea wood-fall micro-ecosystems (61). Although lignocellulolytic bacteria exist, they are not aggressive degraders of wood substrates (62). In this study, fungal communities in the wood fall have the potential to secrete various lignocellulases. For example, important cellulases Endo-β−1,4-glucanase (EC 3.2.1.4) was found in GH45 (63), while the hemicellulases xyloglucan-specific endo-β−1,4-glucanase (EC 3.2.1.151) (64) and Endo-β−1,3-glucanase (EC 3.2.1.39) (65) were identified in GH152. Moreover, the cellooligosaccharide dehydrogenase in AA7 can fuel cellulose degradation by lytic polysaccharide monooxygenase in AA9 (66). Thus, fungi break down lignocellulose in wood fall through the synergistic collaboration of the aforementioned enzymes. Notably, the CE1 from bacterial communities contains acetylxylan esterase (EC 3.1.1.72), an essential hemicellulose degradation enzyme (67, 68). However, the complete degradation of hemicellulose relies on the synergistic action of multiple hemicellulases (69). Therefore, the potential contribution of bacteria to lignocellulose degradation in wood-fall micro-ecosystems is limited.

The species and functional contribution analyses indicated that the fungal communities in wood fall contributed to lignocellulose degradation only in SWCS and TR. In SWCS, Microascaceae contributed 100% to AA9, AA7, and GH45 families containing cellulases, while Nectriaceae contributed 100% to GH152, which contains hemicellulases. Moreover, the cellulose and hemicellulose levels in SWCS were significantly lower than in SDCS and TR. The above results suggest that Microascaceae and Nectriaceae may have the potential to degrade cellulose and hemicellulose effectively in wood-fall micro-ecosystems by secreting a variety of lignocellulases. Previous studies have shown that cellulases are typically produced by saprophytic microorganisms that thrive on dead or decaying organic matter, and certain plant pathogens also possess the ability to express cellulases (70, 71). Most members of the Microascaceae are found in terrestrial environments, primarily acting as saprobic in soil and rotting vegetation (72). Some of these species can act as saprobes or pathogens (73). However, Microascaceae has also been found to exhibit a broader geographic distribution in marine environments, inhabit diverse habitats, and play various ecological roles (74). Studies on Nectriaceae have revealed their widespread presence in various terrestrial environments, including woody substrates and soil (75, 76). Nectriaceae have been identified as plant pathogens (77), causing a variety of symptoms such as fruit rot, canker disease, and dieback disease (78), which can lead to tree death in severe cases (79). Additionally, Song et al. (80) further discovered that Nectriaceae are capable of breaking down cellulose and lignin in soil, probably due to the presence of genes encoding both cellulose- and lignin-degrading enzymes (81). Nevertheless, our results indicate that Nectriaceae possess genetic potential for cellulase production in the deep-sea environment as well.

In summary, this study revealed that lignocellulose degradation in wood-fall micro-ecosystems is potentially mediated by fungi. Fungal communities in wood fall appear to possess the genetic potential to secrete a wide range of lignocellulases, in contrast to the limited potential of bacteria. Moreover, the terrestrial fungi Microascaceae and Nectriaceae may efficiently degrade cellulose and hemicellulose in deep-sea wood fall through the secretion of diverse lignocellulases.

### Conclusion

This study found that all other physicochemical indices differed significantly among different wood-fall contact surfaces, except TS. Among them, TC and TN contents were significantly higher in SDCS than in TR, whereas cellulose and hemicellulose contents were significantly lower in SWCS than in SDCS and TR, suggesting that cellulose and hemicellulose were effectively degraded in SWCS of wood fall. The SDCS was considered a high-carbon environment, whereas the TR was a potentially nitrogen-limited area. Notably, the bacterial and fungal community structures differed significantly among distinct wood-fall contact surfaces. Thus, the habitat heterogeneity of distinct contact surfaces may influence the structure of microbial communities and lays the foundation for environmental filtering to shape microbial community assembly in deep-sea wood-fall micro-ecosystems. The community assembly results showed that dispersal limitation and homogenizing dispersal only occurred in SWCS and TR, affecting the community assembly processes of bacterial and fungal communities, respectively. Additionally, the Venn diagram visualization revealed that the shared fungal community between SWCS and TR was predominantly composed of Aspergillaceae, which have strong dispersal capabilities and might inhibit the growth and dispersal of bacteria by secreting secondary metabolites with bacteriostatic activities. These findings suggest that environmental filtering and organism exchange levels regulate the assembly of wood-fall microbiomes. Functional analysis showed that bacteria displayed a higher metabolic potential for sulfur, nitrogen, and methane metabolism. Functional profiles associated with dissimilatory nitrate reduction and denitrification were dominant among distinct contact surfaces, suggesting that both closed and open nitrogen cycling existed in wood-fall micro-ecosystem. In addition, dissimilatory sulfate reduction and methanogenesis were enriched in SDCS, whereas TR-enriched processes related to nitrate reduction. The above results indicate that bacteria may play an important role in sulfur, nitrogen, and methane metabolism in wood-fall micro-ecosystems, potentially contributing to biogeochemical cycling through diverse metabolic pathways. However, under nitrogen-limited conditions, bacteria tend to participate in closed nitrogen cycles. Moreover, fungi exhibit greater genetic potential for secreting lignocellulases than bacteria, suggesting that lignocellulose degradation in wood-fall micro-ecosystems is primarily mediated by fungi. The terrestrial fungi Microascaceae and Nectriaceae may efficiently degrade both cellulose and hemicellulose in wood-fall micro-ecosystems through the synergistic action of multiple lignocellulases. Accordingly, our study illustrates the microbiome dynamics and functional profiles of distinct contact surfaces in wood fall and provides new insights into the driving pattern of community assembly, biogeochemical processes, and lignocellulose degradation in deep-sea wood-fall micro-ecosystems. Furthermore, this study advances our understanding of the ecological impacts of organic falls on deep-sea oligotrophic environments.

## MATERIALS AND METHODS

### Sample collection

Cruise TS2-10-2 was carried out by the R/V *Tansuo 2* in the SCS in December 2021. During a dive of the human-occupied vehicle “*Shenhaiyongshi*” (Dive SY420, December 23, 2021), a natural deep-sea wood fall was found at a depth of 2321.6 m on the northern slope of the Zhongnan seamount in the SCS (Fig. 1a) and collected using a remotely operated vehicle (ROV). To avoid the loss of organisms, the wood log was put by the ROV manipulator into a separate box closed with a lid. The entire wood-fall sample (length: 125 cm, width: 38 cm, height: 14 cm; 115°30′5.76″ E; 14°01′9.95″ N) was subdivided into SDCS, SWCS, and TR, which had different signs of decay (Fig. 1b and c). Sixteen wood chip samples were cut from the distinct contact surfaces (four from SWCS, four from SDCS, and eight from TR) on board the ship using sterilized tools and transferred to 50 mL cryotubes in liquid nitrogen. Any visible organisms (macro- and megafauna) were removed from the wood chips, and wood samples were stored at −80°C until DNA extraction.

### Physicochemical indices measurement of wood fall

The samples were dried in 101–4A digital blast drying oven (BOZHEN, China) at 80°C until a consistent weight was achieved. Once dried, they were ground using a pulverizer, passed through a 40 mesh sieve, and sealed for preservation. The cellulose, hemicellulose, and lignin contents of wood-fall samples were extracted and detected using the Cellulose Content Assay Kit (JC0415-M, JC DTECT, China), Hemicellulose Content Assay Kit (JC0416-M, JC DTECT, China), and Lignin Content Assay Kit (BC4205, Solarbio, China), respectively, following the methods described in the kits’ instructions. Cellulose content was determined using an anthrone chromogenic agent under strong acidic conditions. Hemicellulose is converted into reducing sugars after acid treatment, forming a reddish-brown substance with 3,5-dinitrosalicylic acid, exhibiting a characteristic absorption peak at 540 nm, then the absorbance value reflects the hemicellulose content. The phenolic hydroxyl groups in lignin have a characteristic absorption peak at 280 nm after acetylation, and the light absorption value at 280 nm is positively correlated with the lignin content. Therefore, wavelengths of 620, 540, and 280 nm were, respectively, used for the cellulose, hemicellulose, and lignin content measurements via Infinite 200 Pro M Nano (Tecan Trading AG, Switzerland). Distilled water (for cellulose and hemicellulose) and glacial acetic acid (for lignin) were used as blank controls to measure absorbance and determine the content of cellulose, hemicellulose, and lignin. The Kjeldahl method was used to determine the TN content. The TC and TS contents were measured using an Elemental Analyzer (Vario EL Cube, Hanau, Germany).

### DNA extraction, amplicon, and metagenome sequencing

Prior to DNA extraction, each wood sample was homogenized using a FastPrep-24 5G instrument (MP Biomedicals, CA, USA). Total microbial DNA was extracted and purified from the wood powder with the FastDNA Spin Kit for Soil (MP Biomedicals, CA, USA) following the manufacturer’s protocol. Then the concentration and purity of the extracted DNA were determined with TBS-380 (Turner BioSystems, Sunnyvale, CA, USA) and NanoDrop 2000 (Thermo-Fisher, Waltham, MA, USA) fluorometry and spectrophotometry, respectively, and quality was checked by 1% agarose gel electrophoresis. Only high-quality DNA was selected as the template for PCR amplification. The samples were sequenced using an Illumina System (Majorbio Bio-Pharm Technology Co. Ltd., Shanghai, China). For the amplicon sequencing, the hypervariable region of the bacterial 16S rRNA gene (V3–V4 region) was amplified with the primer pairs 338F (5′-ACTCCTACGGGAGGCAGCAG-3′) and 806R (5′-GGACTACHVGGGTWTCTAAT-3′) (82, 83). Meanwhile, the primer pairs ITS3F (5′-GCATCGATGAAGAACGCAGC-3′) and ITS4R (5′-TCCTCCGCTTATTGATATGC-3′) (84) were used to amplify the fungal ITS2 region. PCR products were purified using the AxyPrep DNA Gel Extraction Kit (Axygen Biosciences, Union City, CA, USA) according to the manufacturer’s instructions and quantified using the QuantiFluor -ST system (Promega, USA). Amplicons were pooled at equimolar concentrations, and paired-end sequencing (2 × 300 bp) was performed on the Illumina MiSeq platform (Illumina, San Diego, CA, USA). For metagenome sequencing, the extracted DNA was fragmented to an average size of approximately 350 bp using the ultrasonicator Covaris M220 (Gene Company Limited, China) for paired-end library construction. A paired-end library was constructed using the NEXTFLEX Rapid DNA-Seq Kit (Bioo Scientific, Austin, TX, USA). Adapters containing the full complement of the sequencing primer hybridization sites were ligated to the blunt ends of the fragments. Paired-end sequencing was performed on Illumina NovaSeq PE150 platform (Illumina Inc., San Diego, CA, USA) using NovaSeq Reagent Kits (Illumina, San Diego, CA, USA) according to the manufacturer’s instructions (www.illumina.com).

### 16S rRNA gene and ITS2 sequences analysis

Microbiome sequences were processed using Quantitative Insights Into Microbial Ecology 2 (QIIME 2, v2022.2). After rigorous quality control and filtration using the denoise-paired method in the DADA2 algorithm, the bacterial and fungal reads were clustered into ASVs. The SILVA 138/16S bacteria and Unite 8.0/ITS fungi databases were used to annotate the ASVs of bacteria and fungi, respectively. Sequences assigned to chloroplasts and mitochondria were removed. The β-nearest taxon index (βNTI) and the Bray-Curtis-based Raup-Crick metric (RCbray) were used to determine contributions from deterministic and stochastic processes (34). |βNTI| > 2 indicates that the deterministic process shapes the microbiome community, in which βNTI > 2 denotes heterogeneous selection while βNTI < –two denotes homogeneous selection. A βNTI value less than 2 and greater than −2 indicates a stochastic process that affects the community. The fractions of |βNTI| < 2 were differentiated by the RCbray value. A value of RCbray > 0.95 indicates that the observed turnover is dominated by the dispersal limitation, RCbray < –0.95 indicates homogenizing dispersal, and |RCbray| < 0.95 indicates the effect of drift.

### Metagenomic assembly, annotations, and data processing

Fastp (https://github.com/OpenGene/fastp, version 0.23.0) was used for quality control. Sequencing was performed using MEGAHIT (https://github. com/voutcn/megahit, version 1.1.2). All sequences with an identity cutoff of 95% were clustered, and redundancy was reduced to construct a nonredundant gene set using CD-HIT (http://www.bioinformatics.org/cd-hit/, version 4.6.1) (85). Using SOAPaligner software (https://github.com/ShujiaHuang/SOAPaligner, version 2.21), the high-quality reads of each sample were compared to the nonredundant gene set to determine information on the abundance of genes in the corresponding samples (86). The amino acid sequences of the non-redundant gene sets were compared to the non-redundant (NR) database using Diamond (https://github.com/bbuchfink/diamond, version 0.8.35, blastp, e-value <1e^−5^), and the taxonomic information corresponding to the NR library database was used to obtain species annotations (87). Then, the abundance of the species was calculated using the sum of gene abundances corresponding to the species. The amino acid sequences of the non-redundant gene sets were aligned with the KEGG (https://www.genome.jp/kegg) (88) database using Diamond (blastp, e-value <1e^−5^) for functional annotation. The information on KO groups was obtained through function genes annotations using KOBAS 2.0 software (89). The carbohydrate-active enzyme (CAZymes) annotation was performed using hmmscan (v3.1b2, e-value <1e^−5^, https://www.ebi.ac.uk/Tools/hmmer/search/hmmscan) based on the carbohydrate-active enzyme database (http://www.cazy.org/) (90). The abundance of a module or CAZymes family was calculated by summing the abundances of the corresponding genes. For further analysis, the functional gene sets of bacterial and fungal, including sulfur, nitrogen, methane metabolism, and carbohydrate degradation, were constructed based on the results of NR, KEGG, and CAZy annotations, respectively. To investigate the role of microbial communities in driving the sulfur, nitrogen, and methane cycles in deep-sea wood-fall micro-ecosystem, we analyzed the composition of sulfur, nitrogen, and methane modules among different contact surfaces of wood fall. In this study, we focused on the sulfur, nitrogen, and methane metabolism though annotation of KEGG modules, which represent a functional unit and is a collection of multiple KO numbers (https://www.kegg.jp/kegg/module.html). A KEGG module is considered present as long as 1 KO genes has been identified within it (gene count ≥2; Tables S3 and S4). To establish a potential link between microbial carbohydrate metabolism and the degradation rate of wood falls, we analyzed the alpha diversity of CAZyme genes and the composition of families that containing lignocellulases among different contact surfaces of wood fall. To explore the relative contribution of bacteria and fungi (at family level) to the enriched modules and lignocellulases family, the taxonomic information for each functional gene sets was extracted, and their relationships were calculated using the method described by Ofek-Lalzar et al. (91). All analyses were performed on marker abundances normalized to reads per kilobase per million reads.

### Statistical analysis

Taxonomic alpha diversity was estimated by calculating the Shannon diversity index. Based on R, the Kruskal-Wallis tests were used to determine the differences in the taxonomic alpha diversity and physicochemical indices among distinct contact surfaces of wood fall (significance level threshold of 0.05), and a post hoc test was performed using the Tukey-Kramer tests. Using the vegan package in R (version 3.3.1), PCoA analysis based on the Bray-Curtis distance was performed to visualize changes in the community structure among samples. PERMANOVA was performed to test for significant differences in microbial structure among distinct contact surfaces of wood fall. Venn diagram visualization was performed using the Venn Diagram package to analyze the common numbers of bacteria and fungi at the family level between the SWCS and TR of the wood fall. To investigate the differences in sulfur, nitrogen, and methane metabolism among different contact surfaces of wood fall, the enrichment characteristics of the KEGG modules were analyzed using LEfSe (LDA > 4.0, *P* < 0.05) (92). Then, we evaluated the completeness of the enriched modules. KEGG module completeness was evaluated based on the total number of blocks in a module, the KOs required for each block, and the KOs present in each genome (Tables S3 and S4). The enriched module is considered complete only when all blocks have corresponding KO genes (Fig. S2). The alpha diversity of CAZymes genes was estimated by calculating the Shannon diversity index. The Kruskal-Wallis test was used to determine the differences in the CAZyme genes alpha diversity among distinct contact surfaces of wood fall (significance level threshold of 0.05), and a post hoc test was performed using the Nemenyi tests.

## Data Availability

All sequencing data associated with this project were deposited in the NCBI Sequence Read Archive database (accession numbers: PRJNA1088981, PRJNA1088984, and PRJNA1090054).

## SUPPLEMENTAL MATERIAL

Supplemental figures aem.02165-24-s0001.pdf Figures S1 and S2.

Supplemental tables aem.02165-24-s0002.xlsx Tables S1 to S7.

ASM does not own the copyrights to Supplemental Material that may be linked to, or accessed through, an article. The authors have granted ASM a non-exclusive, world-wide license to publish the Supplemental Material files. Please contact the corresponding author directly for reuse.

## References

[1] West AJ, Lin C-W, Lin T-C, Hilton RG, Liu S-H, Chang C-T, Lin K-C, Galy A, Sparkes RB, Hovius N. 2011. Mobilization and transport of coarse woody debris to the oceans triggered by an extreme tropical storm. Limnol Oceanogr 56:77–85. doi:10.4319/lo.2011.56.1.0077

[2] Mao L, Comiti F. 2010. The effects of large wood elements during an extreme flood in a small tropical basin of Costa Rica. WIT Trans Eng Sci 67:225–236. doi:10.2495/DEB100191

[3] Hamdan LJ, Hampel JJ, Moseley RD, Mugge RL, Ray A, Salerno JL, Damour M. 2021. Deep-sea shipwrecks represent island-like ecosystems for marine microbiomes. ISME J 15:2883–2891. doi:10.1038/s41396-021-00978-y 33888864 PMC8443566

[4] Miettinen J, Shi C, Liew SC. 2011. Deforestation rates in insular Southeast Asia between 2000 and 2010. Glob Chang Biol 17:2261–2270. doi:10.1111/j.1365-2486.2011.02398.x

[5] Bienhold C, Pop Ristova P, Wenzhöfer F, Dittmar T, Boetius A. 2013. How deep-sea wood falls sustain chemosynthetic life. PLoS ONE 8:e53590. doi:10.1371/journal.pone.0053590 23301092 PMC3534711

[6] Jørgensen BB, Boetius A. 2007. Feast and famine--microbial life in the deep-sea bed. Nat Rev Microbiol 5:770–781. doi:10.1038/nrmicro1745 17828281

[7] Smith Craig R, De Leo FC, Bernardino AF, Sweetman AK, Arbizu PM. 2008. Abyssal food limitation, ecosystem structure and climate change. Trends Ecol Evol 23:518–528. doi:10.1016/j.tree.2008.05.002 18584909

[8] Fagervold SK, Galand PE, Zbinden M, Gaill F, Lebaron P, Palacios C. 2012. Sunken woods on the ocean floor provide diverse specialized habitats for microorganisms. FEMS Microbiol Ecol 82:616–628. doi:10.1111/j.1574-6941.2012.01432.x 22703298

[9] Smith CR, Baco AR. 2003. Ecology of whale falls at the deep-sea ﬂoor. In Oceanography and marine biology. CRC Press.

[10] Baco AR, Smith CR. 2003. High species richness in deep-sea chemoautotrophic whale skeleton communities. Mar Ecol Prog Ser 260:109–114. doi:10.3354/meps260109

[11] Pop Ristova P, Bienhold C, Wenzhöfer F, Rossel PE, Boetius A. 2017. Temporal and spatial variations of bacterial and faunal communities associated with deep-sea wood falls. PLoS One 12:e0169906. doi:10.1371/journal.pone.0169906 28122036 PMC5266260

[12] Kalenitchenko D, Le Bris N, Dadaglio L, Peru E, Besserer A, Galand PE. 2018. Bacteria alone establish the chemical basis of the wood-fall chemosynthetic ecosystem in the deep-sea. ISME J 12:367–379. doi:10.1038/ismej.2017.163 28984846 PMC5776450

[13] Cunha MR, Matos FL, Génio L, Hilário A, Moura CJ, Ravara A, Rodrigues CF. 2013. Are organic falls bridging reduced environments in the deep sea? - results from colonization experiments in the Gulf of Cádiz. PLoS ONE 8:e76688. doi:10.1371/journal.pone.0076688 24098550 PMC3788751

[14] Amon DJ, Hilario A, Arbizu PM, Smith CR. 2017. Observations of organic falls from the abyssal Clarion-Clipperton Zone in the tropical eastern Pacific Ocean. Mar Biodiv 47:311–321. doi:10.1007/s12526-016-0572-4

[15] Bernardino AF, Smith CR, Baco A, Altamira I, Sumida PYG. 2010. Macrofaunal succession in sediments around kelp and wood falls in the deep NE Pacific and community overlap with other reducing habitats. Deep-Sea Res I: Oceanogr Res Pap 57:708–723. doi:10.1016/j.dsr.2010.03.004

[16] Duperron S, Laurent MCZ, Gaill F, Gros O. 2008. Sulphur-oxidizing extracellular bacteria in the gills of Mytilidae associated with wood falls. FEMS Microbiol Ecol 63:338–349. doi:10.1111/j.1574-6941.2008.00438.x 18218025

[17] Levine JM, HilleRisLambers J. 2009. The importance of niches for the maintenance of species diversity. Nature New Biol 461:254–257. doi:10.1038/nature08251 19675568

[18] Nemergut DR, Schmidt SK, Fukami T, O’Neill SP, Bilinski TM, Stanish LF, Knelman JE, Darcy JL, Lynch RC, Wickey P, Ferrenberg S. 2013. Patterns and processes of microbial community assembly. Microbiol Mol Biol Rev 77:342–356. doi:10.1128/MMBR.00051-12 24006468 PMC3811611

[19] Hubbell SP. 2001. The unified neutral theory of biodiversity and biogeography. Princeton University Press, Princeton. 10.1016/j.tree.2011.03.024 21561679

[20] Chave J. 2004. Neutral theory and community ecology. Ecol Lett 7:241–253. doi:10.1111/j.1461-0248.2003.00566.x

[21] Zhou J, Ning D. 2017. Stochastic community assembly: does it matter in microbial ecology? Microbiol Mol Biol Rev 81:10–1128. doi:10.1128/MMBR.00002-17 PMC5706748 29021219

[22] Kalenitchenko D, Fagervold SK, Pruski AM, Vétion G, Yücel M, Le Bris N, Galand PE. 2015. Temporal and spatial constraints on community assembly during microbial colonization of wood in seawater. ISME J 9:2657–2670. doi:10.1038/ismej.2015.61 25885564 PMC4817635

[23] Palacios C, Zbinden M, Pailleret M, Gaill F, Lebaron P. 2009. Highly similar prokaryotic communities of sunken wood at shallow and deep-sea sites across the oceans. Microb Ecol 58:737–752. doi:10.1007/s00248-009-9538-4 19547939

[24] Kang Q, Appels L, Tan T, Dewil R. 2014. Bioethanol from lignocellulosic biomass: current findings determine research priorities. Sci World J 2014:1–13. doi:10.1155/2014/298153 PMC4295598 25614881

[25] Dos Santos AC, Ximenes E, Kim Y, Ladisch MR. 2019. Lignin-enzyme interactions in the hydrolysis of lignocellulosic biomass. Trends Biotechnol 37:518–531. doi:10.1016/j.tibtech.2018.10.010 30477739

[26] Leschine SB. 1995. Cellulose degradation in anaerobic environments. Annu Rev Microbiol 49:399–426. doi:10.1146/annurev.mi.49.100195.002151 8561466

[27] Mouzouras R, Jones EG, Venkatasamy R, Holt DM. 1988. Microbial decay of lignocellulose in the marine environment, p 343–356. In Marine biodeterioration

[28] Meerts P. 2002. Mineral nutrient concentrations in sapwood and heartwood: a literature review. Ann For Sci 59:713–722. doi:10.1051/forest:2002059

[29] Li J, Dong C, Lai Q, Wang G, Shao Z. 2022. Frequent occurrence and metabolic versatility of Marinifilaceae bacteria as key players in organic matter mineralization in global deep seas. mSystems 7:e0086422. doi:10.1128/msystems.00864-22 36342154 PMC9765461

[30] Horton DE, Johnson NC, Singh D, Swain DL, Rajaratnam B, Diffenbaugh NS. 2015. Contribution of changes in atmospheric circulation patterns to extreme temperature trends. Nature New Biol 522:465–469. doi:10.1038/nature14550 26108856

[31] Cavender-Bares J, Kozak KH, Fine PVA, Kembel SW. 2009. The merging of community ecology and phylogenetic biology. Ecol Lett 12:693–715. doi:10.1111/j.1461-0248.2009.01314.x 19473217

[32] Webb CO, Ackerly DD, McPeek MA, Donoghue MJ. 2002. Phylogenies and community ecology. Annu Rev Ecol Syst 33:475–505. doi:10.1146/annurev.ecolsys.33.010802.150448

[33] McClain CR, Boolukos CM, Bryant SRD, Hanks G. 2023. Sunken trees in the deep sea link terrestrial and marine biodiversity. Ecology 104:e4168. doi:10.1002/ecy.4168 37712249

[34] Stegen JC, Lin X, Fredrickson JK, Konopka AE. 2015. Estimating and mapping ecological processes influencing microbial community assembly. Front Microbiol 6:370. doi:10.3389/fmicb.2015.00370 25983725 PMC4416444

[35] Liu Z, Zhao J-Y, Sun S-F, Li Y, Liu Y-B. 2020. Fungi: outstanding source of novel chemical scaffolds. J Asian Nat Prod Res 22:99–120. doi:10.1080/10286020.2018.1488833 30047298

[36] Li H, Fu Y, Song F. 2023. Marine Aspergillus: a treasure trove of antimicrobial compounds. Mar Drugs 21:277. doi:10.3390/md21050277 37233471 PMC10222851

[37] Xiang Y, Zeng Q, Mai Z-M, Chen Y-C, Shi X-F, Chen X-Y, Zhong W-M, Wei X-Y, Zhang W-M, Zhang S, Wang F-Z. 2021. Asperorydines N-P, three new cyclopiazonic acid alkaloids from the marine-derived fungus Aspergillus flavus SCSIO F025. Fitoterapia 150:104839. doi:10.1016/j.fitote.2021.104839 33513431

[38] Qun T, Zhou T, Hao J, Wang C, Zhang K, Xu J, Wang X, Zhou W. 2023. Antibacterial activities of anthraquinones: structure-activity relationships and action mechanisms. RSC Med Chem 14:1446–1471. doi:10.1039/d3md00116d 37593578 PMC10429894

[39] Zhang J, Duan Y, Bi H, Cai L, Liu L. 2023. Secondary metabolites of the marine-derived Aspergillus jensenii LW128 and the antibacterial activity. Acta Microbiol Sin 63:419–429. doi:10.13343/j.cnki.wsxb.20220360

[40] de Vries RP, Riley R, Wiebenga A, Aguilar-Osorio G, Amillis S, Uchima CA, Anderluh G, Asadollahi M, Askin M, Barry K, et al.. 2017. Comparative genomics reveals high biological diversity and specific adaptations in the industrially and medically important fungal genus Aspergillus. Genome Biol 18:28. doi:10.1186/s13059-017-1151-0 28196534 PMC5307856

[41] Damare S, Raghukumar C, Raghukumar S. 2006. Fungi in deep-sea sediments of the Central Indian Basin. Deep-Sea Res I: Oceanogr Res Pap 53:14–27. doi:10.1016/j.dsr.2005.09.005

[42] Zuluaga-Montero A, Ramírez-Camejo L, Rauscher J, Bayman P. 2010. Marine isolates of Aspergillus flavus: denizens of the deep or lost at sea? Fungal Ecol 3:386–391. doi:10.1016/j.funeco.2010.05.003 21076639 PMC2976972

[43] O’Malley MA. 2008. “Everything is everywhere: but the environment selects”: ubiquitous distribution and ecological determinism in microbial biogeography. Stud Hist Philos Biol Biomed Sci 39:314–325. doi:10.1016/j.shpsc.2008.06.005 18761283

[44] Levin LA, Barry JP, Felbeck H, Smith CR, Young CM. 2007. Advances in vent, seep, whale- and wood-fall biology. Mar Ecol 28:1–2. doi:10.1111/j.1439-0485.2007.00153.x

[45] Wasmund K, Mußmann M, Loy A. 2017. The life sulfuric: microbial ecology of sulfur cycling in marine sediments. Environ Microbiol Rep 9:323–344. doi:10.1111/1758-2229.12538 28419734 PMC5573963

[46] Fors Y, Nilsson T, Risberg ED, Sandström M, Torssander P. 2008. Sulfur accumulation in pinewood (Pinus sylvestris) induced by bacteria in a simulated seabed environment: implications for marine archaeological wood and fossil fuels. Int Biodeterior Biodegradation 62:336–347. doi:10.1016/j.ibiod.2007.11.008

[47] Muyzer G, Stams AJM. 2008. The ecology and biotechnology of sulphate-reducing bacteria. Nat Rev Microbiol 6:441–454. doi:10.1038/nrmicro1892 18461075

[48] Reeburgh WS, Heggie DT. 1977. Microbial methane consumption reactions and their effect on methane distributions in freshwater and marine environments. Limnol Oceanogr 22:1–9. doi:10.4319/lo.1977.22.1.0001

[49] Winfrey MR, Zeikus JG. 1977. Effect of sulfate on carbon and electron flow during microbial methanogenesis in freshwater sediments. Appl Environ Microbiol 33:275–281. doi:10.1128/aem.33.2.275-281.1977 848951 PMC170678

[50] Oremland RS, Taylor BF. 1978. Sulfate reduction and methanogenesis in marine sediments. Geochim Cosmochim Acta 42:209–214. doi:10.1016/0016-7037(78)90133-3

[51] Senior E, Lindström EB, Banat IM, Nedwell DB. 1982. Sulfate reduction and methanogenesis in the sediment of a saltmarsh on the East coast of the United kingdom. Appl Environ Microbiol 43:987–996. doi:10.1128/aem.43.5.987-996.1982 16346022 PMC244174

[52] Conrad R. 2009. The global methane cycle: recent advances in understanding the microbial processes involved. Environ Microbiol Rep 1:285–292. doi:10.1111/j.1758-2229.2009.00038.x 23765881

[53] Ermler U, Grabarse W, Shima S, Goubeaud M, Thauer RK. 1997. Crystal structure of methyl-coenzyme M reductase: the key enzyme of biological methane formation. Science 278:1457–1462. doi:10.1126/science.278.5342.1457 9367957

[54] Kraft B, Tegetmeyer HE, Sharma R, Klotz MG, Ferdelman TG, Hettich RL, Geelhoed JS, Strous M. 2014. Nitrogen cycling. The environmental controls that govern the end product of bacterial nitrate respiration. Science 345:676–679. doi:10.1126/science.1254070 25104387

[55] Marchant HK, Lavik G, Holtappels M, Kuypers MMM. 2014. The fate of nitrate in intertidal permeable sediments. PLoS ONE 9:e104517. doi:10.1371/journal.pone.0104517 25127459 PMC4134218

[56] Orsi WD, Edgcomb VP, Christman GD, Biddle JF. 2013. Gene expression in the deep biosphere. Nature New Biol 499:205–208. doi:10.1038/nature12230 23760485

[57] Cochrane VW. 1958. Physiology of fungi. John Wiley & Sons, Hoboken.

[58] Obeng EM, Adam SNN, Budiman C, Ongkudon CM, Maas R, Jose J. 2017. Lignocellulases: a review of emerging and developing enzymes, systems, and practices. Bioresour Bioprocess 4:16. doi:10.1186/s40643-017-0146-8

[59] Andlar M, Rezić T, Marđetko N, Kracher D, Ludwig R, Šantek B. 2018. Lignocellulose degradation: an overview of fungi and fungal enzymes involved in lignocellulose degradation. Eng Life Sci 18:768–778. doi:10.1002/elsc.201800039 32624871 PMC6999254

[60] Hyde KD, Jones EBG, Leaño E, Pointing SB, Poonyth AD, Vrijmoed LLP. 1998. Role of fungi in marine ecosystems. Biodivers Conserv 7:1147–1161. doi:10.1023/A:1008823515157

[61] Dupont J, Magnin S, Rousseau F, Zbinden M, Frebourg G, Samadi S, Forges BR, Jones EG. 2009. Molecular and ultrastructural characterization of two ascomycetes found on sunken wood off Vanuatu Islands in the deep Pacific Ocean. Mycol Res 113:1351–1364. doi:10.1016/j.mycres.2009.08.015 19737615

[62] Holt DM, Jones EB. 1983. Bacterial degradation of lignified wood cell walls in anaerobic aquatic habitats. Appl Environ Microbiol 46:722–727. doi:10.1128/aem.46.3.722-727.1983 6639026 PMC239341

[63] Annamalai N, Rajeswari MV, Balasubramanian T. 2016. Endo-1, 4-β-glucanases: role, applications and recent developments, p 37–45. In Microbial enzymes in bioconversions of biomass. doi:10.1007/978-3-319-43679-1_3.

[64] Grishutin SG, Gusakov AV, Markov AV, Ustinov BB, Semenova MV, Sinitsyn AP. 2004. Specific xyloglucanases as a new class of polysaccharide-degrading enzymes. Biochim Biophys Acta 1674:268–281. doi:10.1016/j.bbagen.2004.07.001 15541296

[65] Gavande PV, Goyal A. 2023. Chapter 6 - endo-β-1,3-glucanase. In Glycoside hydrolases. Academic Press, San Diego.

[66] Haddad Momeni M, Fredslund F, Bissaro B, Raji O, Vuong TV, Meier S, Nielsen TS, Lombard V, Guigliarelli B, Biaso F, Haon M, Grisel S, Henrissat B, Welner DH, Master ER, Berrin J-G, Abou Hachem M. 2021. Discovery of fungal oligosaccharide-oxidising flavo-enzymes with previously unknown substrates, redox-activity profiles and interplay with LPMOs. Nat Commun 12:2132. doi:10.1038/s41467-021-22372-0 33837197 PMC8035211

[67] Nakamura AM, Nascimento AS, Polikarpov I. 2017. Structural diversity of carbohydrate esterases. Biotechnol Res Innov 1:35–51. doi:10.1016/j.biori.2017.02.001

[68] Kim M-J, Jang M-U, Nam G-H, Shin H, Song J-R, Kim T-J. 2020. Functional expression and characterization of acetyl xylan esterases CE family 7 from Lactobacillus antri and Bacillus halodurans. J Microbiol Biotechnol 30:155–162. doi:10.4014/jmb.2001.01004 31986559 PMC9728288

[69] Payne CM, Knott BC, Mayes HB, Hansson H, Himmel ME, Sandgren M, Ståhlberg J, Beckham GT. 2015. Fungal cellulases. Chem Rev 115:1308–1448. doi:10.1021/cr500351c 25629559

[70] Juturu V, Wu JC. 2014. Microbial cellulases: engineering, production and applications. Renew Sustain Energy Rev 33:188–203. doi:10.1016/j.rser.2014.01.077

[71] Hwang IS, Oh E-J, Lee HB, Oh C-S. 2019. Functional characterization of two cellulase genes in the Gram-positive pathogenic bacterium Clavibacter michiganensis for wilting in tomato. Mol Plant Microbe Interact 32:491–501. doi:10.1094/MPMI-08-18-0227-R 30345870

[72] Sakayaroj J, Pang K-L, Jones EBG. 2011. Multi-gene phylogeny of the Halosphaeriaceae: its ordinal status, relationships between genera and morphological character evolution. Fungal Divers 46:87–109. doi:10.1007/s13225-010-0072-y

[73] Sandoval-Denis M, Gené J, Sutton DA, Cano-Lira JF, Hoog GS, Decock CA, Wiederhold NP, Guarro J. 2016. Redefining Microascus, Scopulariopsis and allied genera, p 1–36. In Persoonia - molecular phylogeny and evolution of fungi. Vol. 36. doi:10.3767/003158516X688027. PMC4988368 27616786

[74] Wang M-M, Yang S-Y, Li Q, Zheng Y, Ma H-H, Tu Y-H, Li W, Cai L. 2024. Microascaceae from the marine environment, with descriptions of six new species. J Fungi 10:45. doi:10.3390/jof10010045 PMC10821522 38248952

[75] Lombard L, van der Merwe NA, Groenewald JZ, Crous PW. 2015. Generic concepts in Nectriaceae. Stud Mycol 80:189–245. doi:10.1016/j.simyco.2014.12.002 26955195 PMC4779799

[76] Zeng Z-Q, Zhuang W-Y. 2022. New species of Nectriaceae (Hypocreales) from China. J Fungi 8:1075. doi:10.3390/jof8101075 PMC9605599 36294639

[77] Savary O, Coton M, Jany J-L, Coroller L, Coton E. 2022. Effect of abiotic factors and culture media on the growth of cheese-associated Nectriaceae species. Int J Food Microbiol 364:109509. doi:10.1016/j.ijfoodmicro.2021.109509 35030441

[78] Perera RH, Hyde KD, Jones EBG, Maharachchikumbura SSN, Bundhun D, Camporesi E, Akulov A, Liu JK, Liu ZY. 2023. Profile of Bionectriaceae, Calcarisporiaceae, Hypocreaceae, Nectriaceae, Tilachlidiaceae, Ijuhyaceae fam. nov., Stromatonectriaceae fam. nov. and Xanthonectriaceae fam. nov. Fungal Divers 118:95–271. doi:10.1007/s13225-022-00512-1

[79] Guarnaccia V, Kraus C, Markakis E, Alves A, Armengol J, Eichmeier A, Compant S, Gramaje D. 2022. Fungal trunk diseases of fruit trees in Europe: pathogens, spread and future directions. Phytopathol Mediterr 61:563–599. doi:10.36253/phyto-14167

[80] Song A, Zhang J, Xu D, Wang E, Bi J, Asante-Badu B, Njyenawe MC, Sun M, Xue P, Wang S, Fan F. 2022. Keystone microbial taxa drive the accelerated decompositions of cellulose and lignin by long-term resource enrichments. Sci Total Environ 842:156814. doi:10.1016/j.scitotenv.2022.156814 35732237

[81] Wilhelm RC, Singh R, Eltis LD, Mohn WW. 2019. Bacterial contributions to delignification and lignocellulose degradation in forest soils with metagenomic and quantitative stable isotope probing. ISME J 13:413–429. doi:10.1038/s41396-018-0279-6 30258172 PMC6331573

[82] Huse SM, Dethlefsen L, Huber JA, Mark Welch D, Relman DA, Sogin ML. 2008. Exploring microbial diversity and taxonomy using SSU rRNA hypervariable tag sequencing. PLoS Genet 4:e1000255. doi:10.1371/journal.pgen.1000255 19023400 PMC2577301

[83] Mori H, Maruyama F, Kato H, Toyoda A, Dozono A, Ohtsubo Y, Nagata Y, Fujiyama A, Tsuda M, Kurokawa K. 2014. Design and experimental application of a novel non-degenerate universal primer set that amplifies prokaryotic 16S rRNA genes with a low possibility to amplify eukaryotic rRNA genes. DNA Res 21:217–227. doi:10.1093/dnares/dst052 24277737 PMC3989492

[84] White T, Bruns T, Lee S, Taylor J, Innis M, Gelfand D, Sninsky J. 1990. Amplification and direct sequencing of fungal ribosomal RNA genes for phylogenetics. Academic Press, Diego.

[85] Fu L, Niu B, Zhu Z, Wu S, Li W. 2012. CD-HIT: accelerated for clustering the next-generation sequencing data. Bioinformatics 28:3150–3152. doi:10.1093/bioinformatics/bts565 23060610 PMC3516142

[86] Li R, Yu C, Li Y, Lam T-W, Yiu S-M, Kristiansen K, Wang J. 2009. SOAP2: an improved ultrafast tool for short read alignment. Bioinformatics 25:1966–1967. doi:10.1093/bioinformatics/btp336 19497933

[87] Buchfink B, Xie C, Huson DH. 2015. Fast and sensitive protein alignment using DIAMOND. Nat Methods 12:59–60. doi:10.1038/nmeth.3176 25402007

[88] Kanehisa M, Goto S. 2000. KEGG: Kyoto encyclopedia of genes and genomes. Nucleic Acids Res 28:27–30. doi:10.1093/nar/28.1.27 10592173 PMC102409

[89] Xie C, Mao X, Huang J, Ding Y, Wu J, Dong S, Kong L, Gao G, Li C-Y, Wei L. 2011. KOBAS 2.0: a web server for annotation and identification of enriched pathways and diseases. Nucleic Acids Res 39:W316–W322. doi:10.1093/nar/gkr483 21715386 PMC3125809

[90] Lombard V, Golaconda Ramulu H, Drula E, Coutinho PM, Henrissat B. 2014. The carbohydrate-active enzymes database (CAZy) in 2013. Nucleic Acids Res 42:D490–D495. doi:10.1093/nar/gkt1178 24270786 PMC3965031

[91] Ofek-Lalzar M, Sela N, Goldman-Voronov M, Green SJ, Hadar Y, Minz D. 2014. Niche and host-associated functional signatures of the root surface microbiome. Nat Commun 5:4950. doi:10.1038/ncomms5950 25232638

[92] Segata N, Izard J, Waldron L, Gevers D, Miropolsky L, Garrett WS, Huttenhower C. 2011. Metagenomic biomarker discovery and explanation. Genome Biol 12:R60. doi:10.1186/gb-2011-12-6-r60 21702898 PMC3218848

