# Supplementary material for: Microbiome dynamics and functional profiles in deep-sea wood-fall micro-ecosystem: insights into drive pattern of community assembly, biogeochemical processes, and lignocellulose degradation
Source: Appl Environ Microbiol. 2024 Dec 6;91(1):e02165-24. doi: 10.1128/aem.02165-24 (PMC11784029; doi:10.1128/aem.02165-24)
Supplement: Supplemental figures — Figures S1 and S2. [file aem.02165-24-s0001.pdf]

Supplementary information for:

Microbiome dynamics and functional profiles in deep-sea wood-fall micro-ecosystem: insights into drive pattern of community assembly, biogeochemical processes and lignocellulose degradation

Zeming Bao[a], Biao Chen[a*], Kefu Yu[a,b], Yuxin Wei[a], Xinyue Liang[a], Huanting Yao[a], Xianrun Liao[a], Wei Xie[b,c], Kedong Yin[b,c]

[a] *Guangxi Laboratory on the Study of Coral Reefs in the South China Sea; Coral Reef Research Center of China; School of Marine Sciences, Guangxi University, Nanning, China*

[b] *Southern Marine Science and Engineering Guangdong Laboratory (Zhuhai), Zhuhai, 519082, China*

[c] *School of Marine Sciences, Sun Yat-sen University, Zhuhai, 519082, China*

Running title: Deep-sea wood-fall microbial ecology

Corresponding author:

[*] Corresponding Author

Dr. Biao Chen Email: biaochenwork@163.com

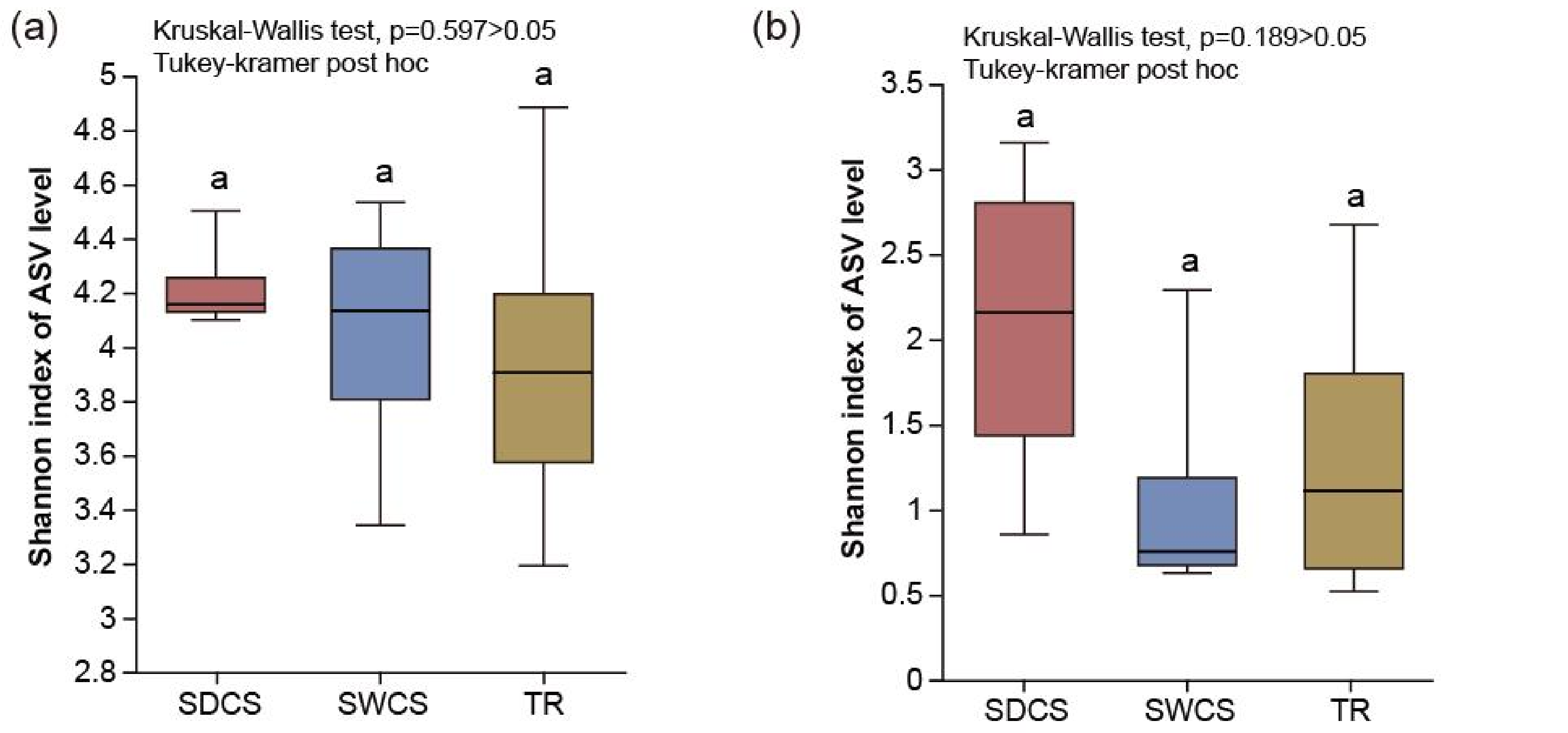

Figure S1. The alpha diversity of bacterial and fungal community among distinct contact surfaces of deep-sea wood fall. There was no significantly differences of Shannon diversity index for (a) bacterial and (b) fungal community among distinct contact surfaces of wood fall. Kruskal-Wallis test.

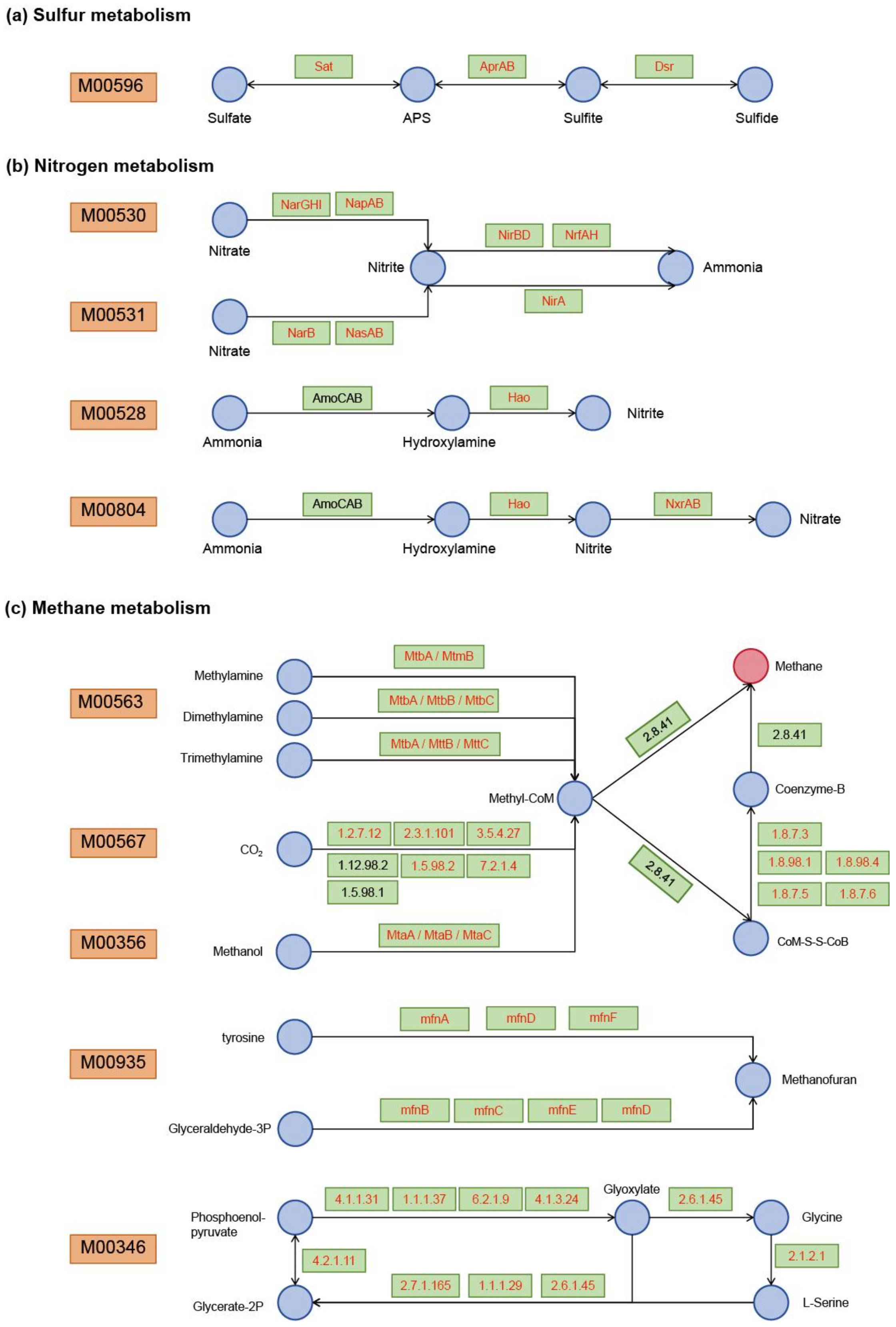

Figure S2. Metabolic pathway map of enriched modules. (a) sulfur metabolism; (b) nitrogen metabolism; (c) methane metabolism. The red font indicates the functional gene or Enzyme detected in the bacterial genome. A functional gene name or Enzyme ID may correspond to multiple KOs.
